# Impact of the COVID-19 pandemic on antidepressant use in eleven European regions: a comparative time series analysis 2018–2022

**DOI:** 10.1007/s00127-025-02962-9

**Published:** 2025-07-22

**Authors:** Iva Selke Krulichová, Adam Hallberg, Gisbert W. Selke, Katri Aaltonen, Manuela Casula, Jurij Fürst, Katarina Gvozdanović, Mohammadhossein Hajiebrahimi, Amanj Kurdi, Fredrik Nyberg, Elena Olmastroni, Hanna Rättö, Juraj Slabý, Björn Wettermark, Tanja Mueller

**Affiliations:** 1 Faculty of Medicine in Hradec Králové, Department of Medical Biophysics, Charles University, Hradec Králové, Czech Republic; 2 Pharmacoepidemiology & Social Pharmacy, Department of Pharmacy, Uppsala University, Uppsala, Sweden; 3 AOK Research Institute (WIdO), Berlin, Germany; 4 INVEST Research Centre, University of Turku, Turku, Finland; 5 The Social Insurance Institution of Finland, Helsinki, Finland; 6 Epidemiology and Preventive Pharmacology Service (SEFAP), Department of Pharmacological and Biomolecular Sciences, University of Milan, Milan, Italy; 7 IRCCS MultiMedica, Sesto San Giovanni, Milan, Italy; 8 Health Insurance Institute, Ljubljana, Slovenia; 9 Teaching Institute of Public Health “Dr. Andrija Stampar”, Zagreb, Croatia; 10 Strathclyde Institute of Pharmacy and Biomedical Sciences, University of Strathclyde, Glasgow, UK; 11 College of Pharmacy, Al-Kitab University, Kirkuk, Iraq; 12 Department of Public Health Pharmacy and Management, School of Pharmacy, Sefako Makgatho Health Sciences University, Pretoria, South Africa; 13 School of Public Health and Community Medicine, Institute of Medicine, Sahlgrenska Academy, University of Gothenburg, Gothenburg, Sweden; 14 State Institute for Drug Control, Prague, Czech Republic; 15 Pharmacy Center, Department of Medicine, Vilnius University, Vilnius, Lithuania

**Keywords:** Antidepressants, Mental health, COVID-19, Cross-national comparison, Interrupted time series analysis, Drug utilization research

## Abstract

**Purpose:**

The COVID-19 pandemic had detrimental effects on the mental health of populations, with differing influences on different demographic groups. Varying national countermeasures to the pandemic may have further impacted these effects. This study aimed to explore the effects of the pandemic on dispensed volumes of antidepressants in outpatient settings in different regions of Europe and to assess potential age- and sex-related differences of its impact on incidence of antidepressant dispensing.

**Methods:**

We used descriptive and interrupted time series analyses of pharmacy dispensing data on volumes. For six regions, we analysed volume and incident use stratified by age and sex.

**Results:**

During the pandemic, the preexisting long-term trend in unstratified dispensed volumes significantly increased only in Slovenia and Germany and weakened in Scotland and Wales (estimated changes in slope + 0.16, + 0.10, − 0.23, and − 0.68 defined daily doses per thousand inhabitants per day, respectively, for each month). The stratified quarterly analysis revealed the greatest relative increase in females aged 0–17 (+ 64% in Sweden to + 167% in Croatia in the last quarter of 2022 compared with the last quarter of 2019). Both rate of change and difference between sexes were lower in higher age groups. Incidence increased most steeply in females aged 0–17, where the estimated pandemic-related increase explained 11% (Sweden) to 55% (Lombardy) of new patients receiving antidepressants.

**Conclusion:**

Our findings indicate the need to develop targeted mental health supporting measures to increase resilience, especially in young people, and mitigate the impact of potential future public health crises.

**Supplementary Information:**

The online version contains supplementary material available at 10.1007/s00127-025-02962-9.

## Introduction

The COVID-19 pandemic, declared by the World Health Organization (WHO) on 11 March 2020 and ending on 5 May 2023 [1], has caused at least 7 million deaths globally [2] and has placed immense strain on people’s health and well-being. In addition to the direct effects of the disease on physical health, the uncertainty regarding the disease and its long-term effects on health, well-being and the private economy also had detrimental effects on mental health; moreover, many countries implemented restrictions on social distancing and limited movements of people, which may have exacerbated this further [3, 4]. In addition, implemented restrictions may have negatively affected access to healthcare [5]. However, the approaches of different countries to the pandemic differed greatly. Sweden, for example, never experienced any major impact of heavy mandatory measures, such as a lockdown, on the general functioning of society, and the availability of psychological health care was shown in a previous study to be relatively unaffected [6]; in contrast, the UK experienced several complete lockdowns, with severe restrictions imposed on people’s daily lives, including limited availability of healthcare services, particularly those affecting specialist services, including mental health care [7].

The increasing prevalence of mental health conditions as a topic of concern predates the COVID-19 pandemic but has gained further attention since, not least because many health services are already struggling to cope with various public health threats, including alarming rates of antimicrobial resistance and increasing levels of noncommunicable diseases such as cancer [8, 9]. A meta-analysis published in 2020 highlighted a prevalence of depression and anxiety of ~ 34% and ~ 32%, respectively, in the countries surveyed, which included (amongst others) Italy, Spain, and the UK [10]. Subsequently, a systematic review on mental health in Europe during the COVID-19 pandemic indicated a higher prevalence of several mental health conditions, including depression, during the pandemic than during prior periods [11]; similarly, an umbrella review published in 2023 reported a significant increase in depression in the general population across many countries in the first year of the pandemic, as well as a small but significant increase in symptoms of anxiety [12]. Furthermore, there is reason to assume that there are differences in susceptibility to pandemic-related mental health issues in subgroups of the population, such as higher vulnerability in children and adolescents [13, 14], the elderly [13], or women [15].

Nevertheless, evidence on the specific effect of the COVID-19 pandemic on depression incidence and prevalence trends remains inconclusive, as research findings differ widely depending on study design, country, and time period studied [4, 11, 16]. Potentially, this has been aggravated by methodological challenges related to the diagnosis and recording of mental health conditions, particularly during the pandemic, where access to services may have been negatively impacted, possibly resulting in underdiagnosis of depression among populations. Consequently, there has been a drive to use prescribing and/or dispensing data on antidepressants as a proxy for diagnoses, although the findings of these studies have also been inconclusive thus far. For example, in the United States, a significant increase in anxiety and depression medication use was reported in March and April 2020 [17]. In contrast, studies conducted in Italy and Sweden reported no immediate obvious effect on prescribing [6, 18], whereas in Canada, an initial decrease in prescribing early in the pandemic was followed by an increase [19]. These delayed effects on prescribing and the discrepancies between prescribing and self-reported diagnoses of depression might be attributable to a number of reasons but could include stockpiling of medication [18]. A general challenge when comparing studies across countries are differences in study designs, measurement units, and populations studied. Direct comparative cross-national studies applying the same methodology, including countries taking different approaches during the pandemic, would therefore add value.

### Study aim

The primary aim of this study was to explore dispensed volumes of antidepressants prescribed in the outpatient sector between 2018 and 2022 in different regions of Europe to gain better insight into the changes that have occurred during the COVID-19 pandemic. The secondary aim was to assess potential age- and sex-related differences in the impact of the pandemic on the incidence of antidepressant dispensing.

## Methods

### Study design and data

We conducted a retrospective, observational, cross-national comparative study in eleven European countries/regions of different geographic locations, health systems, and administrative responses to the pandemic. The participating countries and regions were Croatia, Czechia, Finland, Germany, Lombardy (Italy), Slovenia, Sweden, Northern Ireland, England, Scotland, and Wales; for general country information, see the Online Resource, Table S1.

For each country/region, we used monthly pharmacy dispensing data from January 2018 to December 2022. The data included all dispensed prescription antidepressants (defined as daily doses (DDDs)) designated as N06A in the Anatomical-Therapeutic-Chemical (ATC) classification defined by the WHO [20]. For six countries/regions (Croatia, Finland, Germany, Lombardy, Slovenia, Sweden), the data were stratified into five age groups (0–17, 18–44, 45–64, 65–74 and 75 years and over) and by sex. In addition, for these six countries/regions, monthly data on counts of incident recipients of antidepressants were provided. Incident recipients within a month were defined as those who had not received any antidepressant in the previous 12 months. The details on the data sources are provided in the Online Resource, Table S2.

### Statistical analysis

We analysed dispensed volumes and the incidence of dispensing antidepressants. Where possible, we used stratification by age and sex to gain more detailed insight. We defined the pre-COVID-19 period as January 2018 to February 2020 and the COVID-19 period as March 2020 to December 2022. Data analysis was performed using R, version 4.4.0 [21].

### Study outcomes

For the purpose of comparing the volumes of dispensed antidepressants across periods and countries/regions, we expressed the volume of the dispensed DDDs in each period (month or quarter) as DDD/TID (i.e., the number of dispensed DDDs per thousand inhabitants per day). We used the following equation:

$$\:{v}_{p}=\frac{{\sum\:}_{m=1}^{k}{u}_{m,p}}{{d}_{p}{n}_{p}}\cdot\:1000$$

*v*_*p*_: dispensed volume in period *p* [DDD/TID]. *u*_*m, p*_: dispensed volume in month *m* of period *p* [DDD]. *k*: number of months in period *p*. *d*_*p*_: number of days in period *p*. *n*_*p*_: size of population covered in period *p*.

We calculated the treatment incidence in each period (month or quarter) as the number of incident recipients per 100,000 persons (of the same age group and sex). We used this equation:

$$\:{incidence}_{p}=\frac{\sum\:{k}_{p}}{\sum\:{n}_{p}}\cdot\:100000$$

*incidence*_*p*_: incidence in period *p*. *k*_*p*_: number of incident patients. *n*_*p*_: size of population (in the same age and sex group) covered in period *p*.

### Dispensed volumes

Using unstratified data, we performed a quarterly analysis of relative changes, which enabled us to compare the percentage changes in the COVID-19 period with those in the pre-COVID-19 period. This was complemented with an interrupted time series analysis of monthly data, which enabled us to estimate the impact of the pandemic. To describe development in sex and age strata, we used quarterly analysis of relative changes.

To assess relative changes in volume, we took the prepandemic year 2019 as the base year and compared the dispensed volume (DDD/TID) in each quarter of the other years with the dispensed volume in the corresponding quarter of 2019. We expressed the relative change as a percentage and used the following formula:

$$\:\varDelta\:{v}_{q,y}=\left(\frac{{v}_{q,y}}{{v}_{q,2019}}-1\right)\cdot\:100$$

Δ*v*_*q, y*_: relative change between quarter *q* in year *y* and the same quarter in 2019 [%]. *v*_*q, y*_: dispensed volume in quarter *q* of year *y* [DDD/TID].

### Interrupted time series analysis of monthly changes in DDD/TID

We used interrupted time series analysis based on autoregressive integrated moving average (ARIMA) models [22] with external regressors [23] to model antidepressant use before and after the onset of the pandemic, allowing us to assess both immediate and delayed changes, thus providing insight into the impact of the pandemic. The use of ARIMA models allowed us to account for seasonality and autocorrelation in the data. We considered 60 monthly time points, 26 in the pre-COVID-19 period and 34 in the COVID-19 period. (For details of the models, cf. the Online Resource.)

We introduced two types of external regressors (i.e., additional variables). To capture the short-term changes in the first three months of the pandemic, we introduced a regressor that reflected the presumed stockpiling in March 2020 and the consecutive decrease in dispensed amounts in April and May 2020. In Slovenia, we replaced this regressor with one modelling a decrease in March and April 2020 followed by an increase in May 2020 to model the dispensing pattern observed for this country in a previous study [24]. To assess the longer-term effect of the pandemic, we explored changes in trend. For this purpose, we used a ramp regressor [23] with onset immediately subsequent to the short-term regressor, i.e., in June 2020, and finishing at the end of the observation period, i.e., December 2022. To assess the quality of the models, we used the Ljung-Box test with a maximum lag of 12 on the residuals to test for the presence of remaining autocorrelation and the Kwiatkowski-Phillips-Schmidt-Shin test for trend stationarity and level stationarity of the residuals. Statistical significance of coefficients in the models was tested using the z-test. Tests were performed at the 0.05 significance level.

### Incidence

For the six countries/regions in which stratified data were available, we calculated the quarterly incidence for each age and sex group. For each region and sex in the group with the most marked quarterly relative changes (youngest age group), we performed, in addition, an interrupted time series analysis of monthly incidence to obtain more detailed insight and to estimate to what degree the observed changes could be attributed to the COVID-19 pandemic.

To assess relative changes in quarterly incidence, we used 2019 as the base year and compared the incidence in each quarter of the other years with the incidence in the corresponding quarter of 2019. We used the following formula:

$$\:\varDelta\:{incidence}_{q,y}=\left(\frac{{incidence}_{q,y}}{{incidence}_{q,2019}}-1\right)\cdot\:100$$

Δ*incidence*_*q, y*_: change in incidence in quarter *q* of year *y* relative to the same quarter in 2019 [%]. *incidence*_*q, y*_: incidence in quarter *q* of year *y*.

### Interrupted time series analysis of monthly incidence in the age group 0–17 years

We also used interrupted time series analysis with ARIMA modelling to analyse the impact of the pandemic on the monthly incidence in the age group 0–17 years. We introduced three regressors. The first of them was to capture the expected temporary decline in incidence in the first three months of the pandemic, as this period was accompanied by extensive lockdowns in many countries. The second and third regressors aimed to capture changes in trend. The second regressor was designed as a ramp from June 2020 to December 2021 and maintained the December 2021 level throughout 2022 (the latter to capture a hypothesized moderation of dynamics due to easing of anti-COVID-19 measures). The third regressor was a ramp to reflect any possibly remaining trend in 2022. The quality of the model was assessed in the same way as described above for DDD/TID. (See the Online Resource for more detail.)

## Results

### Dispensed total volume

**Fig. 1:**
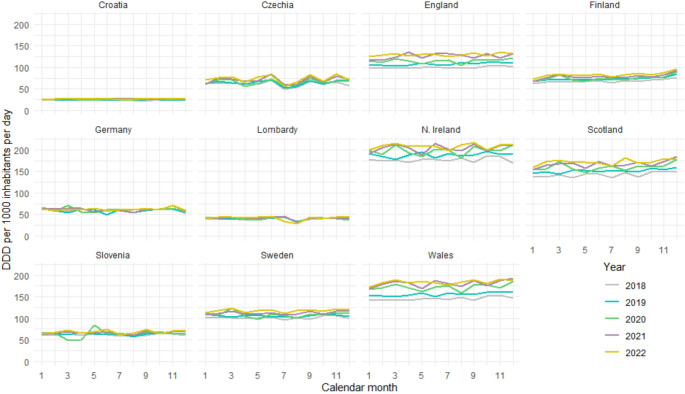
Development of antidepressants dispensing in DDD per 1000 inhabitants per day (DDD/TID) on a monthly scale for 11 European countries/regions from January 2018 to December 2022

Throughout the study period, the highest dispensed volumes (DDD/TID) of antidepressants were observed in Northern Ireland, Wales, Scotland, and England (reaching approximately 200, 180, 170, and 130 DDD/TID, respectively, in 2022). The lowest values were observed in Croatia and Lombardy, with DDD/TID values less than 30 and 50, respectively (Fig. 1).

**Fig. 2:**
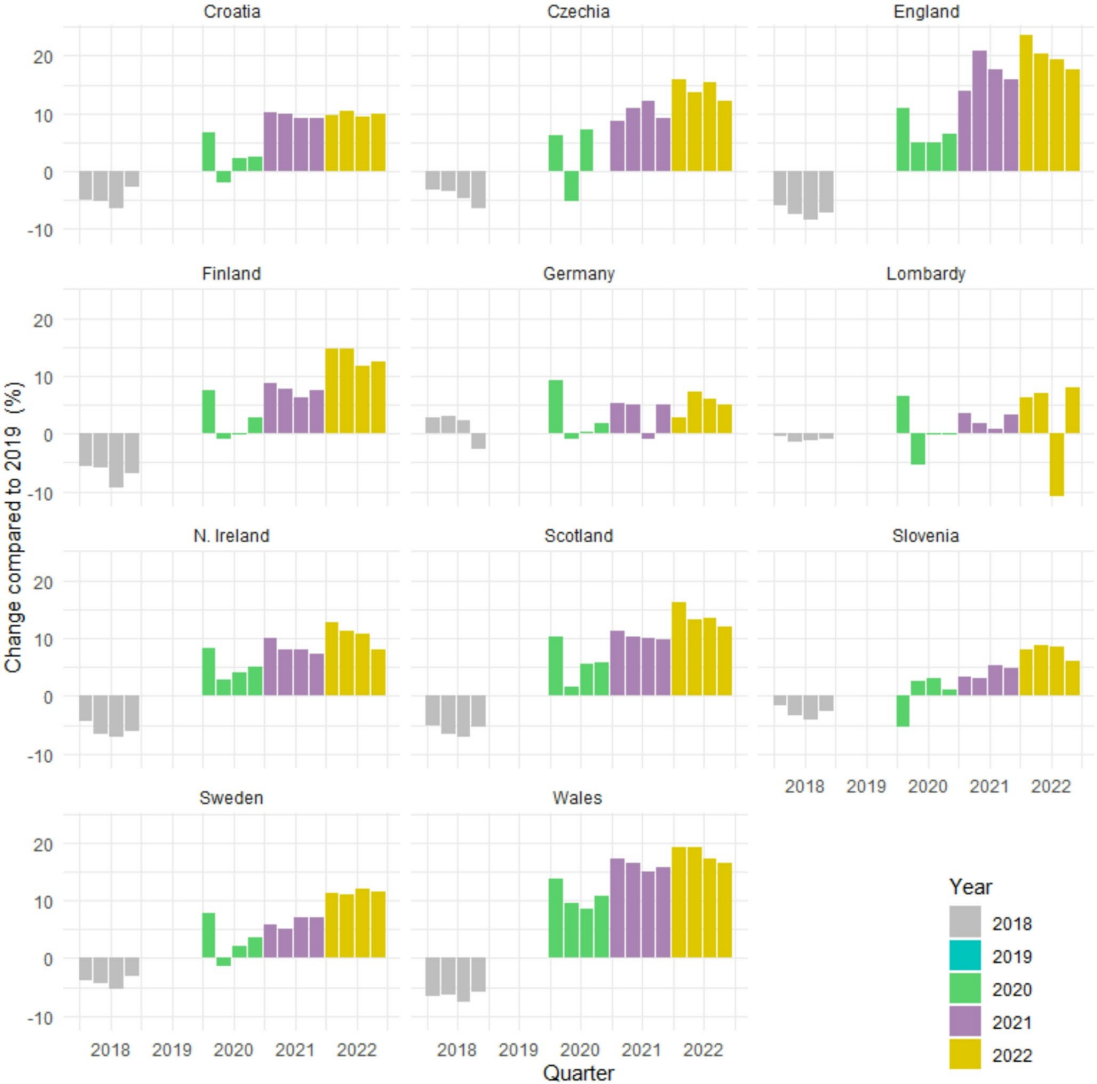
Quarterly changes (%) in dispensed volumes of antidepressants (DDD/TID) from 2018 to 2022 relative to the corresponding quarters of 2019

The quarterly analysis of relative changes revealed that during the entire observation period, the volume of dispensed antidepressants gradually increased in almost all countries/regions. The largest increases relative to 2019 were in England and Wales, with values of up to 24% and 19% in 2022, respectively (Fig. 2). However, the dispensed volumes in these countries had been quickly growing already before the pandemic.

ARIMA modelling of monthly DDD/TID data showed that the regressor modelling the short-term effect in the first three months of the pandemic (increase in March 2020 and decrease in April and May 2020; in Slovenia, decrease in March and April 2020 and increase in May 2020) was significant for all countries/regions. (Cf. notes below Table S3 in the Online Resource for an interpretation.) The regressor for the gradual, permanent effect of the pandemic was significant only in the models for four countries: in Germany and Slovenia, its values indicated a slight increase compared with the pre-COVID trend. In Scotland and Wales, the values implied a decrease in the trend slope during the COVID-19 period, i.e., volumes still rose but not as quickly as before the pandemic. For the last month of the observation period (December 2022), the estimated pandemic-related change in dispensed DDD/TID ranged from − 11.2% (Wales) to + 10.0% (Slovenia) of the observed value (Table 1). The details of the models (estimates of the coefficients, their standard errors, and p-values) are presented in Table S3 and Figures S1–S11 in the Online Resource.

**Table 1:** Estimated pandemic-related changes in volume of dispensed antidepressants at the end of the observation period (December 2022)

					Pandemic-related change in 12/2022 (DDD/TID)			
Country/ region	Observed value in 12/2022 (DDD/TID)	Estimated monthly rate of pandemic- related change (DDD/TID/month)	95% confidence interval (DDD/TID/month)		Middle estimate	Low estimate	High estimate	Share of middle estimate on observed value (%)
Croatia	27.420	–0.008	–0.071	0.055	–	–	–	–
Czechia	70.706	0.798	–0.866	2.462	–	–	–	–
England	131.300	0.019	–0.789	0.827	–	–	–	–
Finland	95.824	0.239	–0.173	0.651	–	–	–	–
Germany	58.070	0.101	0.050	0.152	3.131	1.551	4.711	5.4
Lombardy	44.483	0.025	–0.108	0.158	–	–	–	–
N. Ireland	211.000	–0.293	–0.605	0.019	–	–	–	–
Scotland	176.800	–0.225	–0.311	-0.139	–6.975	–9.648	–4.302	–3.9
Slovenia	70.229	0.227	0.154	0.300	7.037	4.789	9.285	10.0
Sweden	119.187	0.064	–0.136	0.264	–	–	–	–
Wales	188.200	–0.678	–1.335	-0.021	–21.018	–41.373	–0.663	–11.2

Note: Estimates of pandemic-related change in 12/2022 were calculated only for statistically significant monthly rates of pandemic-related change

### Dispensed volumes stratified by age and sex

The most pronounced relative quarterly changes during the COVID-19 period compared with 2019 occurred in the 0–17 age group, particularly among females. In females, the changes were most notable in countries/regions with low pre-COVID antidepressant dispensing rates (up to + 166% in Croatia and + 156% in Lombardy in 2022). For males in this age group, the increase was more moderate: it was most pronounced in Finland and Croatia (up to + 51% and + 49% in 2022 compared with 2019, respectively). An interesting situation occurred in Lombard males, where all quarters of 2020 were below the 2019 level. However, this was followed by an increase that reached more than 40% at the end of 2022. (See the Online Resource, Fig. S12 for more details.)

In women in the age group 18–44, dispensed DDD/TID were throughout higher than in the youngest age group; however, the increase relative to 2019 was less than 30% during the pandemic. In men, the increase was even less marked and did not exceed 15%. In Lombard males, the changes in the COVID-19 period ranged from − 11% to + 6% (Online Resource, Fig. S13).

In all three older age groups, the situation was even more stable, with changes relative to 2019 not exceeding ± 16% for both women and men (Online Resource, Fig. S14–S16). Notably, in Swedish men aged 65–74 years, the quarterly DDD/TID of dispensed antidepressants were lower than in 2019 starting from the second quarter of 2020 until the end of the COVID-19 period, whereas in the highest age group (75+ years), the dispensed volume increased (up to 13% in 2022).

### Incidence by age and sex

A comparison of the prepandemic incidence across age groups showed that the incidence was higher in higher age groups. However, there were exceptions to this rule. Although in all countries/regions, the lowest incidence was in the youngest age group, in Finland and Sweden, the 18–44 age group had the second highest incidence. In Finland, during the pandemic, the incidence in women in this age group even surpassed the incidence in the oldest age group. On the other hand, in Croatian men, the highest incidence before the COVID-19 pandemic was in the age group 45–64, whereas during the pandemic, the oldest age group took the lead (see the Online Resource, Fig. S17–S21 for details).

### Quarterly incidence relative to 2019

In the age group 0–17 years, the most marked changes were observed in Lombardy, where the incidence in 2022 was more than twofold that of 2019 in males and up to fourfold in females. In contrast, in German males of this age group, the incidence remained below the 2019 level in the majority of quarters during the observation period, gradually returning to the 2019 level (Online Resource, Fig. S22, S23).

For the other groups, the relative changes were more moderate. After an initial decrease in 2020, the incidence gradually returned to or exceeded the levels reported in 2019. The observed changes ranged from − 39% (Croatian males, 45–64 years, 2nd quarter of 2020) to + 24% (Slovenian women, 18–44 years, last quarter of 2021). (Online Resource, Fig. S22–S31).

### Monthly incidence in the age group 0–17 years

Since the analysis of quarterly data showed that the most marked changes in incidence after the outbreak of the COVID-19 pandemic occurred in the age group 0–17 years, especially in females, we performed in addition an interrupted time series analysis on monthly data for this age group. It showed that in all countries/regions except Sweden, the average incidence in the first three months of the pandemic was significantly reduced in both males and females. In Sweden, a significant decrease was observed only in females. In the following period (June 2020 to December 2021), we observed a significant increase in trend for females in all countries/regions. For males, an increase in trend was observed in Croatia, Finland, Lombardy, and Sweden, whereas in Germany and Slovenia, the change in trend was not significant. In 2022, the average monthly incidence remained at approximately the level of the end of 2021. The exceptions were Lombardy and Slovenia. In Lombardy, the incidence in males accelerated further in 2022 (*p* < 0.001); in females, the positive trend for 2022 approached statistical significance (*p* = 0.063). In contrast, in Slovenia, we observed a significant downward trend in female incidence in 2022 (*p* < 0.001). The estimates for the pandemic-related changes in incidence explain between 11.0% (Sweden) and 55.4% (Lombardy) of the total incidence in females aged 0–17 years over the whole pandemic period (Table 2). See the Online Resource (Table S4, Fig. S32–S43) for details on the estimates of the coefficients, their standard errors, p-values, interpretation of the estimated values of the coefficients, and fitted models.

**Table 2:** Incidence in the age group 0–17 years: estimated cumulative change associated with the COVID-19 pandemic in the period 3/2020 to 12/2022

		Observed incidence per 100,000 in the period 3/2020–12/2022	Cumulative change of incidence per 100,000 associated with the pandemic in 3/2020–12/2022			
Country/region	Sex		Middle estimate	Low estimate	High estimate	Share of middle estimate on observed incidence (%)
Croatia	Female	953.9	361.2	242.4	479.9	37.9
	Male	373.0	61.2	–5.6	127.9	16.4
Finland	Female	3275.9	815.7	404.8	1226.6	24.9
	Male	1031.4	71.1	–27.6	169.8	6.9
Germany	Female	1200.9	154.0	62.2	245.9	12.8
	Male	552.8	–11.1	–19.3	–2.9	–2.0
Lombardy	Female	533.6	295.8	217.0	374.6	55.4
	Male	200.5	65.3	34.3	96.2	32.5
Slovenia	Female	873.0	182.2	30.2	334.1	20.9
	Male	248.8	–8.9	–17.4	–0.3	–3.6
Sweden	Female	2353.4	259.5	120.5	398.5	11.0
	Male	1180.1	92.8	55.9	129.7	7.9

Note: Low and high estimates are based on lower resp. upper limits of the 95% confidence intervals of statistically significant estimated values of each of the three external regressors of the ARIMA models. The range from low to high accumulated estimates thus may include 0.

## Discussion

In this cross-national comparative study including eleven European regions, we investigated the impact of the COVID-19 pandemic on dispensed volumes of antidepressants from the beginning of the pandemic until the end of 2022. In addition, we assessed the impact of the pandemic on the incidence of dispensed antidepressants stratified by age and sex where available. We observed some common patterns among the regions but also substantial differences among age and sex strata. Overall, the greatest changes were observed in children and adolescents. Nevertheless, our findings using a consistent methodology to investigate relative changes in prescribing support the notion that the effect of COVID-19 on the use of antidepressants varied widely across Europe. This may reflect the influence of many factors, such as the stringency of pandemic-induced measures [25], restrictions on social contacts [25, 26], access to mental health services, pandemic-related mortality, vaccination rates, economic challenges [26], or the prevalence of depression before the pandemic.

### Key findings

#### March 2020 to May 2020: the initial phase of the pandemic

In all countries/regions except Slovenia, there was an immediate increase in dispensed volumes when the pandemic started in March 2020, followed by a decrease below the expected level in the following two months. This pattern may indicate hoarding of medicines at the very beginning of the pandemic and subsequent use of the stores during the first wave of lockdowns or other restrictive measures. In contrast, in Slovenia, we observed a decrease in March and April followed by a peak in May 2020, i.e., after the end of the first lockdown. These findings are in line with the patterns observed in previous studies [24, 27].

The detailed analysis of incidence in children and adolescents, where the greatest changes were observed, revealed a decrease below the expected level in the initial phase of the pandemic, which is in accordance with findings of Gyllenberg et al. [28], Yunus et al. [29], and Chai et al. [30], who reported considerably reduced use of specialist mental health services in children and adolescents in the spring of 2020. However, the occurrence of symptoms of depression and anxiety in vulnerable patients, including young people, has been reported to have increased substantially during the initial lockdowns in most countries. For example, in Czechia, increases in symptoms of depression and anxiety have been reported for the first phase of the COVID-19 pandemic between April and June 2020 [31]; similarly, increased incidences of depression and anxiety disorders have been observed in children and adolescents after the first phase of the COVID-19 pandemic in Finland [28]. In Italy, the overall prevalence of depressive symptoms increased from 33.6% before to 38.9% after the first lockdown [32]; the national-level prevalence of depressive and anxiety symptoms doubled, affecting more than one third of the general adult population and increasing the use of at least one psychotropic drug [33]. In contrast, an overall reduction in depressive symptoms has been reported in Germany in the first year of the pandemic (April 2020 to January 2021) compared with the previous year [34]; nevertheless, while mental well-being was largely unchanged during lockdowns in the elderly population in Germany, depression and anxiety increased among young people, particularly among individuals with no preexisting mental health conditions [35].

This discrepancy between the observed incidence/volume of dispensing of antidepressant medication and reported mental health symptoms suggests undertreatment of mental health conditions during this period, which might, at least partially, be attributed to reduced access to mental health services during the COVID-19 pandemic. In the UK, for example, there was a substantial decrease in mental health service referrals and contacts between April and August 2020 compared with the same period in the preceding year [36].

#### June 2020 to December 2022: longer-term development during the pandemic

We found interesting differences between countries/regions in the longer-term trends in dispensed volumes of antidepressants; for example, there was a slight increase compared with the pre-COVID-19 trend in Germany and Slovenia, whereas in Scotland and Wales, the trend was still positive but lower than that before the pandemic. The most marked percentage changes relative to 2019 were observed in the age group 0–17 years, especially in females in countries/regions with low prepandemic dispensing, such as Croatia and Lombardy, where the dispensed volumes more than doubled during the pandemic. In males, the increase was within 50%, which confirms findings that young females are more affected than males [37, 38]. For comparison, the second most affected age group were persons aged 18–44 years, where the changes in all countries/regions did not exceed 30% in women and 15% in men. In the oldest age group (75 + years), which is also considered vulnerable, the increase relative to that in the pre-COVID-19 year of 2019 was within 15% in both males and females. Our findings indicate that the impact of the pandemic on the use of antidepressants was stronger in younger people and that the differences between sexes decreased with increasing age.

In accordance with the stratified volume analysis, the most marked changes in incidence during the later stages of the pandemic were observed in the youngest age group, especially in Lombardy, which belonged to the regions most affected by the COVID-19 pandemic and subsequent restrictive measures. However, an increase attributable to the pandemic, at least in the period until the end of 2021, also occurred in all other countries/regions in this age group, predominantly in females. In 2022, the incidence among the youngest stabilized except for Lombardy, where it continued to grow, and Slovenia, where it started to decrease again. The moderation of dynamics observed in most countries/regions may be a consequence of the easing of anti-COVID-19 measures or of growing resilience.

The trends observed during the pandemic did not always significantly differ from the trends already established before the pandemic. In many countries, incidences of depression (and other mental health issues) and/or the prescription of antidepressants were already on the rise prior to the emergence of COVID-19, with the COVID-19 pandemic just having exacerbated preexisting trends. In Finland, for example, a positive trend in the use of antidepressants predates the pandemic [39]; however, delayed access to services, as well as changes in help-seeking behaviour, have been suggested as explanations for the observed pattern of new diagnoses in children and adolescents during the pandemic [28]. Likewise, mental health challenges had already been present in the UK prior to the COVID-19 pandemic, where a considerable increase in antidepressant prescribing, as well as an increase in the coprescribing of antipsychotics, was already observed among children and adolescents between 2008 and 2018 [40].

### Strengths and limitations

To our knowledge, this is the first wide population-based cross-national study analysing antidepressant use during the COVID-19 pandemic until the end of 2022. Its principal strengths are the long window of observation and cross-national design covering a wide range of European regions with different geographic locations, health systems, and COVID-19-related policies. The long observation period allowed us to separate short- and longer-term effects. The data permitted a breakdown by demographic features, instrumental for describing varied impacts on different subgroups. The analyses include both volume and incidence of dispensed antidepressants, which enables linking and comparing these outcomes. Volumes are in DDD/TID, an internationally comparable unit that takes into account differences in package sizes, formulations, and strengths between and within countries over time. We used consistent methodology and large administrative databases with near-complete data on all antidepressants in the various regions; as such, the study does not suffer from selection or recall bias. The data thus permit identification of commonalities and differences across European regions.

Nevertheless, several limitations should be considered when interpreting the results. First and foremost, the included countries differed considerably in terms of the prevalence of COVID-19 at various times throughout the study period and the nature and stringency of the measures implemented to limit the spread of the disease (e.g., lockdowns), all of which may have affected the help-seeking behaviour of the affected population and the prescribing and dispensing of antidepressants as well as the capacities of the various national healthcare systems. Additionally, we cannot exclude exogenous factors that may have influenced antidepressant use in parallel with the COVID-19 pandemic. For example, Croatia was hit by two strong earthquakes (on March 23rd, 2020, and December 29th, 2020). The effect of the double crisis has not been systematically studied; however, it is expected to have had a deteriorating effect on mental health [41]. Second, data quality and coverage may differ among countries depending on the data source, with subsequent effects on the accuracy of findings. For example, for Finland, the data encompassed all dispensations reimbursed by national health insurance (approximately 91% of all dispensations of medications in ATC class N06 are reimbursed) [42], whereas the Swedish national prescription register data cover both reimbursed and non-reimbursed dispensations [43]. In addition, data stratified by sex and age group were not available for some of the regions included in this study.

## Conclusion

In this study, we assessed the use of antidepressants before and after the outbreak of the COVID-19 pandemic in eleven European countries/regions. We found a substantial set of common developments in these regions, most notably, a marked increase in antidepressant dispensing in female adolescents. Older age groups, including the elderly, were substantially less affected. Also, in higher age groups, COVID-19-associated changes differed much less between males and females. Regions with high antidepressant use already before the pandemic showed no increase in the overall trend of dispensed volume. However, there are also substantial country-, age-, and sex-specific developments. Further research is needed to investigate the effects of various restrictive measures on population subgroups and to find effective targeted measures for increasing resilience and mitigating the impact of potential future public health crises on mental health. Such analysis may require development of a theoretical framework for assessing strategic, pandemic-scale interventions and also detailed patient-level data.

## Electronic supplementary material

Below is the link to the electronic supplementary material.

Supplementary Material 1

## Data Availability

The data and code that support the findings of this study are openly available at https://doi.org/10.5281/zenodo.12803186.
