# Supplementary material for: Impact of the COVID-19 pandemic on antidepressant use in eleven European regions: a comparative time series analysis 2018–2022
Source: Soc Psychiatry Psychiatr Epidemiol. 2025 Jul 22;61(4):701–12. doi: 10.1007/s00127-025-02962-9 (PMC13021844; doi:10.1007/s00127-025-02962-9)
Supplement: Supplementary file 1 — Supplementary Material 1 [file 127_2025_2962_MOESM1_ESM.pdf]

Supplementary material for

Impact of the COVID-19 pandemic on antidepressant use in eleven European regions: a comparative time series analysis 2018–2022

Social Psychiatry and Psychiatric Epidemiology

Iva Selke Krulichová, Adam Hallberg, Gisbert W. Selke, Katri Aaltonen, Manuela Casula, Jurij Fürst, Katarina Gvozdanović, Mohammadhossein Hajiebrahimi, Amanj Kurdi, Fredrik Nyberg, Elena Olmastroni, Hanna Rättö, Juraj Slabý, Björn Wettermark, Tanja Mueller

Corresponding author: Iva Selke Krulichová, Department of Medical Biophysics, Faculty of Medicine in Hradec Králové, Charles University, Hradec Králové, Czech Republic (krulich@lfhk.cuni.cz)

This supplement contains details on data sources, ARIMA models of monthly development in antidepressants dispensing (DDD/TID) for each country/region, quarterly changes (%) in dispensed volumes (DDD/TID) compared to 2019 stratified by age group and sex, monthly incidence stratified by age group and sex, quarterly changes (%) in incidence per 100 000 for 2019–2022 relative to 2019 stratified by age group and sex, and ARIMA models of development in monthly incidence (per 100 000) for the age group 0–17 years.

Table S1: General country information

Country	Health system[a]	Insurance system	Population 2022 [million]	Data coverage	Prevalence of depression in 2019 [cases per 100 000 inhabitants]	Expenditure on antidepressants in 2022 per capita [€]	Percentage of expenditure on antidepressants relative to total expenditure	Co-payment on antidepressants in 2022
Croatia	n/a	Tax-funded national health service, pharmaceutical reimbursements through statutory health insurance	3.9[b]	100.0%	39.7	2.87	0.8%	n/a
Czechia	Supply- and choice-oriented public	Tax-funded national health service, statutory health insurance	10.8[c]	100.0%	36.7	5.85[d]	1.9%[d]	n/a
England	Regulation-oriented public	Tax-funded national health service	53.1[c,e]	100.0%	47.3[f]	4.77	1.2%	0.0%[g]
Finland	Performance and primary-care oriented, public	Tax-funded national health service, statutory health insurance	5.6[b]	100.0%	47.3	10.21	2.4%	58.3%
Germany	Supply- and choice-oriented, public	Statutory health insurance	84.6[c]	87.1%	41.1	8.88	1.2%	11.3%
Lombardy	Regulation-oriented, public	Statutory health insurance	10.0[h]	99.4%	44.6[i]	6.53	1.3%	12.3%
Northern Ireland	Regulation-oriented public	Tax-funded national health service	1.8[c,e]	100.0%	47.3[f]	7.70	2.5%	0.0%
Scotland	Regulation-oriented public	Tax-funded national health service	5.3[c,e]	100.0%	47.3[f]	8.25	2.7%	0.0%
Slovenia	Public with gate-keeping function of the primary care	Statutory health insurance plus on-top co-insurance	2.1[j]	99.7%	41.0	6.02	1.9%	26.5%
Sweden	Performance and primary-care oriented, public	Tax-funded national health service	10.5[k]	100.0%	47.2	6.93	1.4%	52.2%
Wales	Regulation-oriented public	Tax-funded national health service	3.1[c,e]	100.0%	47.3[f]	6.47	3.7%	0.0%

[a] Classification according to Reibling et al. [1]
[b] Population used in calculations: annual values, end of year
[c] Population used in calculations: annual values, mid-year[d] Excludes co-payment
[e] Population as of mid-2021
[f] Value for UK
[g] Prescription charge of £9.90 (as of July 2024) applies per item for non-exempt patients [2]
[h] Population used in calculations: annual values, beginning of year
[i] Value for Italy
[j] Population used in calculations: semi-annual values, start of half-year
[k] Population used in calculations: monthly values

Table S2: Data sources

Region	Dispensed volumes, prevalence and incidence of medicines	General population size	Study population size	Total expenditure	Anti-depressant expenditure	Copayment
Croatia	Central Health Information System (CEZIH) - prescription database operated by Croatian Health Insurance Fund (CHIF). Access to database shared with Croatian Institute for Public Health (CIPH)	Croatian Bureau of Statistics (https://web.dzs.hr/default_e.htm)		Agency for medicinal products and medical devices (HALMED (https://www.halmed.hr/Novosti-i-edukacije/Publikacije-i-izvjesca/Izvjesca-o-potrosnji-lijekova/Izvjesce-o-potrosnji-lijekova-u-Republici-Hrvatskoj-u-2022/)		
Czechia	State Institute for Drug Control – DIS-13 Reporting supplies of distributed human medicinal products	Czech Statistical Office (https://www.czso.cz/csu/czso/obyvatelstvo_hu)		State Institute for Drug Control, internal database – data from insurance companies.		n/a
England	NHS Business Services Authority: Prescription Cost Analysis Monthly Administration Data (https://opendata.nhsbsa.net/dataset/prescription-cost-analysis-pca-monthly-data)	Office for National Statistics (https://www.ons.gov.uk/peoplepopulationandcommunity/populationandmigration/populationestimates/bulletins/annualmidyearpopulationestimates/mid2021)		NHS Business Services Authority (https://www.nhsbsa.nhs.uk/statistical-collections/prescription-cost-analysis-england/prescription-cost-analysis-england-202122)		
Finland	Dispensed medicines reimbursable under the National Health Insurance scheme register, maintained by The Social Insurance Institution of Finland	Statistics Finland (https://pxdata.stat.fi/PxWeb/pxweb/en/StatFin/StatFin__vaerak/statfin_vaerak_pxt_11ra.px/table/tableViewLayout1/)		The Social Insurance Institution of Finland: Kelasto database: Dispensations reimbursable under the NHI scheme (https://tietotarjotin.fi/en/statistical-data/2051231/statistical-database-kelasto)		
Germany	GAmSi and Act*rapid*! databases at AOK Research Institute (WIdO)[a,b]	Statistisches Bundesamt (https://www.destatis.de/DE/Themen/Gesellschaft-Umwelt/Bevoelkerung/Bevoelkerungsstand/Tabellen/liste-zensus-geschlecht-staatsangehoerigkeit.html#651186)	Bundesministerium für Gesundheit, Statistik KM1 (https://www.bundesgesundheitsministerium.de/fileadmin/Dateien/3_Downloads/Statistiken/GKV/Mitglieder_Versicherte/KM1_JD_2022_1_bf.pdf)	Ludwig WD, Mühlbauer B, Seifert R (eds) [3]	AOK Research Institute (WIdO) (unpublished)	
Lombardy	Administrative databases of reimbursed drugs for the Lombardy region	ISTAT (https://demo.istat.it/app/?i=POS&l=en)	Administrative databases of demographics for the Lombardy region	OSMED-AIFA (https://www.aifa.gov.it/en/-/l-uso-dei-farmaci-in-italia-rapporto-osmed-2022)		
Northern Ireland	Health and Social Care Business Services Organisation: GP prescribing data (https://bso.hscni.net/directorates/operations/family-practitioner-services/directorates-operations-family-practitioner-services-information-unit/general-pharmaceutical-services-and-prescribing-statistics/gp-prescribing-data/)	Office for National Statistics (https://www.ons.gov.uk/peoplepopulationandcommunity/populationandmigration/populationestimates/bulletins/annualmidyearpopulationestimates/mid2021)		Northern Ireland Statistics and Research Agency (NISRA) (https://assets.publishing.service.gov.uk/media/649c3299bb13dc0012b2e250/General_Pharmaceutical_Service_Statistics_for_NI_2022-23_-_Report_-_Accessible.pdf)		

Region	Dispensed volumes, prevalence and incidence of medicines	General population size	Study population size	Total expenditure	Anti-depressant expenditure	Copayment
Scotland	Public Health Scotland: Prescriptions in the Community - Datasets - Scottish Health and Social Care Open Data (https://www.opendata.nhs.scot/dataset/prescriptions-in-the-community)	Office for National Statistics (https://www.ons.gov.uk/peoplepopulationandcommunity/populationandmigration/populationestimates/bulletins/annualmidyearpopulationestimates/mid2021)		Public Health Scotland (https://publichealthscotland.scot/publications/dispenser-payments-and-prescription-cost-analysis/dispenser-payments-and-prescription-cost-analysis-financial-year-2021-to-2022/)		
Slovenia	Database at Health Insurance Institute of Slovenia	Statistical Office (https://www.stat.si/StatWeb/news/Index/10268)	Health Insurance Institute of Slovenia (https://www.zzzs.si/novica/obvezno-zdravstveno-zavarovanje-v-letu-2022-obravnava-in-javna-objava-letnega-porocila-zzzs-2022/)	Health Insurance Institute of Slovenia (https://api.zzzs.si/ZZZS/info/egradiva.nsf/0/538c71578f0fd063c125896c0047b208/$FILE/Letno%20porocilo%20ZZZS%202022.pdf)	Health Insurance Institute of Slovenia (https://partner.zzzs.si/zdravila-in-zivila-za-posebne-zdravstvene-namene/podatki-o-porabi-zdravil/)	
Sweden	Prescription dispensing data from the Swedish E-health Agency	Statistics Sweden (https://www.scb.se/en/finding-statistics/statistics-by-subject-area/population/population-composition/population-statistics)	National board of Health and Welfare. Swedish National Prescribed Drug Register (https://www.socialstyrelsen.se/en/statistics-and-data/registers/national-prescribed-drug-register/)	Swedish E-Health Agency (https://www.ehalsomyndigheten.se/yrkesverksam/statistik-och-lake-medelsforsaljning/)		
Wales	NHS Wales Shared Services Partnership: Pharmacy/Practice Dispensing Data (Pharmacy / Practice Dispensing Data - NHS Wales Shared Services Partnership)	Office for National Statistics (https://www.ons.gov.uk/peoplepopulationandcommunity/populationandmigration/populationestimates/bulletins/annualmidyearpopulationestimates/mid2021)		Llywodraeth Cymru – Welsh Government (https://www.gov.wales/primary-care-prescriptions-april-2022-march-2023-html)		

Source for prevalence of depression: Global Burden of Disease Collaborative Network. Global Burden of Disease Study 2019 (GBD 2019) Results [4]

[a] Incomplete data for August and September 2018 were partially imputed.

[b] Data on sex, prevalence and incidence are not comprehensively available and have been estimated based on Act*rapid*! data for citizens insured by AOK.

Notes on autoregressive moving average (ARIMA) modelling

ARIMA models were expressed as ARIMA(p,d,q)(P,D,Q)$_m$, where
p … number of autoregressive parameters (ar) in the model,
d … degree of differencing in the time series (here $d \leq 1$).
q … number of moving average parameters (ma) in the model,
P … number of seasonal autoregressive parameters (sar) in the model,
D … degree of seasonal differencing in the time series (here $D \leq 1$),
Q … number of seasonal moving average parameters (sma) in the model,
m … number of data points (months) in each seasonal cycle (here $m = 12$).

Note: ARIMA models with seasonal effects are also called SARIMA (i.e., seasonal autoregressive moving average) models.

First the time series was made stationary by correcting for trend and/or for seasonality, which was achieved by seasonal and/or non-seasonal differencing. Let Y_t denote the observed value in month t.

Seasonal differencing (for a seasonal cycle of 12 months):

$$Y'_t = \begin{cases} Y_t & \text{if } D = 0 \text{ or } t < 12 \\ Y_t - Y_{t-12} & \text{else} \end{cases}$$

Non-seasonal differencing:

$$Y''_t = \begin{cases} Y'_t & \text{if } d = 0 \text{ or } t < 1 \\ Y'_t - Y'_{t-1} & \text{else} \end{cases}$$

Y''_t denotes the value in month t of the stationary time series after seasonal and/or non-seasonal differencing (if needed). On the stationary time series the autoregressive and moving average parameters were estimated. Having a 12-month seasonal cycle, the values Y''_t of the stationary time series were modelled as follows:.

$$Y''_t = c + \sum_{i=1}^{p} ar_i\, Y''_{t-i} + \sum_{j=1}^{P} sar_j\, Y''_{t-12j} + \sum_{k=1}^{q} ma_k\, \varepsilon_{t-k} + \sum_{l=1}^{Q} sma_l\, \varepsilon_{t-12l} + \varepsilon_t$$

where c is a constant, ar_i and sar_j denote non-seasonal resp. seasonal autoregressive coefficients, ma_k and sma_l denote non-seasonal resp. seasonal moving average coefficients, and ε_t denotes the error term (a random shock occurring at time t).

We added external regressors to assess the effect of the COVID-19 pandemic (see the text below tables S3 and S4).

To determine the appropriate ARIMA model, we used the *auto.arima* function from the *forecast* package in R, where models are evaluated based on Akaike's criterion (AIC) and the model with the lowest AIC value is selected. For the model selected in this way, we tested the statistical significance of the coefficients, and checked whether the model residuals were white noise (Kwiatkowski-Phillips-Schmidt-Shin and Ljung-Box tests). Where needed, we assessed the model by examining the autocorrelation function (ACF) and partial autocorrelation function (PACF) plots and tested the model properties as described above.

Table S3: Details on the ARIMA models used for impact assessment of the pandemic on global dispensed volume of prescription antidepressants (DDD/TID)

Country/region	Model	Parameter	Estimated value	Standard error	p-value†
Croatia	$ARIMA(3,1,1)(0,0,0)_{12}$	ar_1	–0.096	0.119	0.423
		ar_2	–0.0763	0.118	0.519
		ar_3	0.447	0.119	<0.001
		ma_1	–0.999	0.048	<0.001
		drift	0.081	0.019	<0.001
		short-term	2.886	0.654	<0.001
		long-term	–0.008	0.032	0.809
Czechia	$ARIMA(1,1,0)(0,1,0)_{12}$	ar_1	–0.599	0.117	<0.001
		short-term	5.529	1.894	0.004
		long-term	0.798	0.849	0.347
England	$ARIMA(2,1,0)(0,0,0)_{12}$	ar_1	–0.914	0.111	<0.001
		ar_2	–0.494	0.111	<0.001
		drift	–0.612	0.301	0.042
		short-term	6.097	2.408	0.011
		long-term	–0.042	0.418	0.919
Finland	$ARIMA(2,1,0)(0,1,0)_{12}$	ar_1	–0.961	0.099	<0.001
		ar_2	–0.703	0.096	<0.001
		short-term	6.348	0.842	<0.001
		long-term	0.239	0.210	0.255
Germany	$ARIMA(0,0,1)(0,1,1)_{12}$	ma_1	–0.332	0.140	0.018
		sma_1	–0.595	0.222	0.007
		short-term	10.060	2.397	<0.001
		long-term	0.101	0.026	<0.001
Lombardy	$ARIMA(0,1,1)(0,1,0)_{12}$	ma_1	–0.999	0.060	<0.001
		short-term	3.957	1.494	0.008
		long-term	0.025	0.068	0.718
Northern Ireland	$ARIMA(2,1,1)(0,1,0)_{12}$	ar_1	–0.819	0.116	<0.001
		ar_2	–0.636	0.111	<0.001
		ma_1	–0.831	0.107	<0.001
		short-term	11.078	2.754	<0.001
		long-term	–0.293	0.159	0.064
Scotland	$ARIMA(2,1,1)(1,0,0)_{12}$	ar_1	–0.812	0.126	<0.001
		ar_2	–0.494	0.121	<0.001
		ma_1	–0.999	0.035	<0.001
		sar_1	0.663	0.103	<0.001
		drift	0.754	0.028	<0.001
		short-term	17.061	2.125	<0.001
		long-term	–0.225	0.044	<0.001

Country/region	Model	Parameter	Estimated value	Standard error	p-value†
Slovenia	ARIMA(3,0,0)(1,0,0)$_{12}$	ar_1	–0.120	0.123	0.329
		ar_2	–0.089	0.127	0.482
		ar_3	0.301	0.123	0.014
		sar_1	0.424	0.131	0.001
		intercept	62.457	0.617	<0.001
		short-term	23.234	1.739	<0.001
		long-term	0.227	0.037	<0.001
Sweden	ARIMA(2,1,1)(0,1,1)$_{12}$	ar_1	–0.907	0.104	<0.001
		ar_2	–0.725	0.106	<0.001
		ma_1	–0.546	0.143	<0.001
		sma_1	–0.409	0.196	0.037
		short-term	13.200	1.139	<0.001
		long-term	0.0638	0.102	0.534
Wales	ARIMA(2,1,1)(0,1,1)$_{12}$	ar_1	–0.789	0.173	<0.001
		ar_2	–0.573	0.157	<0.001
		ma_1	–0.436	0.247	0.078
		sma_1	–0.627	0.258	0.015
		short-term	11.355	3.361	<0.001
		long-term	–0.678	0.335	0.043

Note: †z-test

Parameters: ar_1 – first order autoregression, ar_2 – second order autoregression, ar_3 – third order autoregression, ma_1 – first order moving average, sar_1 – first order seasonal autoregression, sma_1 – first order seasonal moving average, short-term – short duration effect of the onset of the pandemic in its first three months, long-term – long term effect of the pandemic manifested as change of trend in the period June 2020 to December 2022

For Czechia and Wales, the final model used had a significant Ljung-Box test, which means that residuals were not completely free of autocorrelation, however, we did not manage to find a better parsimonious model.

We introduced two variables to capture the effect of the COVID-19 pandemic on dispensing of antidepressants in DDD/TID. These variables are represented by the parameters "short-term" and "long-term" in the ARIMA models.

The variable capturing the short-term effect of the pandemic had a value of 0 in every month of the study period except for the three months at the beginning of the pandemic (3/2020–5/2020). It had a value of 1 in March 2020 to capture the effect of stockpiling and a value of –0.5 in the following two months as we assumed a lower number of visits to the doctor due to restrictive measures and the use of drugs from stock procured in the previous period. Based on the experience of a previous study [5], we defined this variable differently for Slovenia in the period 3/2020–5/2020 (–0.5 for March and April and 1 for May), because in this country there was first a significant decrease in the dispensing of medicines for chronic diseases and an increase only after the end of the first wave of lock-down in May 2020. The variable capturing the short-term effect of the pandemic is represented by the parameter “short-term” in the above ARIMA models.

The second variable captured long-term change in trend over the period 6/2020–12/2022 and had a value of 0 in the months 1/2018–5/2020 and values of 1–31 in the period 6/2020–12/2022 (in each month the value was 1 higher than in the previous month). This variable is represented by the parameter “long-term”.

The interpretation of the estimates of the pandemic-related parameters is illustrated using Germany as an example:

The estimated value of the parameter "short-term" is 10.060. This value implies that there was an average increase of 10 DDD/TID in March 2020, and an average decrease of 5 DDD /TID (viz., – 0.5 × 10.060) both in April and May 2020.
The estimated value of the parameter "long-term" is 0.101 and significantly different from zero. This value means that, for each month, the number of DDDs dispensed per day per 1000 inhabitants was higher by 0.101, compared to what would have been expected based on the previous month, had there been no effect of the pandemic. Thus, in December 2022 (31st month of the period) the total estimated average increase attributable to the pandemic was 31 × 0.101 = 3.131 DDD/TID, compared to what would have been expected (counterfactually) for this month without the pandemic.

Table S4: Details on the ARIMA models used for impact assessment of the pandemic on monthly incidence of antidepressant patients in the age group 0–17 years

Country/ region	Sex	Model	Parameter	Estimated value	Standard error	p-value†
Croatia	Females	ARIMA(0,0,0)(0,1,0)$_{12}$	short-term	-14.630	2.353	<0.001
			trend21	0.969	0.128	<0.001
			trend22	-0.150	0.223	0.501
	Males	ARIMA(0,0,0)(0,1,0)$_{12}$	short-term	-6.082	1.321	<0.001
			trend21	0.190	0.072	0.008
			trend22	0.003	0.125	0.979
Finland	Females	ARIMA(1,0,3)(0,1,0)$_{12}$	ar_1	0.783	0.111	<0.001
			ma_1	-0.550	0.153	<0.001
			ma_2	-0.286	0.136	0.035
			ma_3	0.568	0.151	<0.001
			short-term	-9.970	2.442	<0.001
			trend21	2.023	0.484	<0.001
			trend22	-0.843	0.641	0.189
	Males	ARIMA(3,0,0)(0,1,0)$_{12}$	ar_1	-0.025	0.141	0.859
			ar_2	-0.169	0.135	0.233
			ar_3	0.317	1.103	0.019
			short-term	-4.445	0.072	<0.001
			trend21	0.202	0.120	0.005
			trend22	-0.097	0.641	0.419
Germany	Females	ARIMA(0,0,0)(0,1,0)$_{12}$	short-term	-7.455	1.831	<0.001
			trend21	0.422	0.099	<0.001
			trend22	-0.296	0.173	0.088
	Males	ARIMA(0,0,0)(0,1,1)$_{12}$	sma_1	-0.709	0.279	0.011
			drift	-0.091	0.041	0.026
			short-term	-3.694	1.393	0.008
			trend21	0.098	0.088	0.266
			trend22	0.135	0.111	0.222
Lombardy	Females	ARIMA(0,1,2)(1,1,0)$_{12}$	ma_1	-0.639	0.169	<0.001
			ma_2	-0.361	0.155	0.020
			sar_1	-0.429	0.141	0.002
			short-term	-2.978	1.282	0.020
			trend21	0.729	0.087	<0.001
			trend22	0.223	0.119	0.062
	Males	ARIMA(0,0,0)(0,1,0)$_{12}$	short-term	-1.538	0.472	0.001
			trend21	0.122	0.026	<0.001
			trend22	0.242	0.045	<0.001
Slovenia	Females	ARIMA(0,0,0)(0,1,0)$_{12}$	short-term	-13.803	2.350	<0.001
			trend21	0.812	0.127	<0.001
			trend22	-1.485	0.223	<0.001
	Males	ARIMA(0,0,0)(0,1,1)$_{12}$	sma_1	-0.690	0.254	0.006
			short-term	-2.959	1.451	0.041
			trend21	0.075	0.050	0.136
			trend22	-0.230	0.127	0.071

Country/ region	Sex	Model	Parameter	Estimated value	Standard error	p-value†
Sweden	Females	ARIMA(0,0,0)(1,1,0)$_{12}$	sar_1	-0.443	0.155	0.004
			drift	0.302	0.078	<0.001
			short-term	-6.852	2.457	0.005
			trend21	0.670	0.152	<0.001
			trend22	-0.058	0.206	0.777
	Males	ARIMA(0,0,0)(0,1,1)$_{12}$	sma1	-0.716	0.353	0.042
			short-term	-1.364	1.314	0.230
			trend21	0.222	0.045	<0.001
			trend22	-0.021	0.117	0.854

Note: †z-test

Parameters: ma_1 – first order moving average, ma_2 – second order moving average, ma_3 – third order moving average, ar_1 – first order autoregression, ar_2 – second order autoregression, ar_3 – third order autoregression, sma*1* – first order seasonal moving average, sar_1 – first order seasonal autoregression, short-term – short duration effect of the onset of the pandemic, trend21 – long term effect manifested as change of trend in the period June 2020 to December 2021 and keeping the level of the last month of 2021 throughout 2022, trend22 – long term effect capturing the trend in 2022.

We introduced three variables to capture the effect of the COVID-19 pandemic on monthly incidence. These variables are represented by the parameters "short-term", "trend21" and "trend22" in the ARIMA models.

The variable capturing the short-term effect of the pandemic had a value of 0 in every month of the study period except the three months at the beginning of the pandemic (3/2020–5/2020), when it had a value of 1.

Incidence in the 0–17 age group showed different long-term trends after the start of the pandemic. In some regions, we observed an increase throughout the period 6/2020–12/2022; in other regions, the increase observed after the initial decline slowed in 2022 or remained at the level of late 2021. In some regions, incidence even declined in 2022.

To capture these different trends, we defined two variables. The first variable (trend21) had a value of 0 in the period 1/2018–5/2020, a value of 1–19 in the period 6/2020–12/2021 (the value increased by 1 each month), and a value of 19 throughout the period 1/2022–12/2022. The second variable (trend22) had a value of 0 in the period 1/2018–12/2021 and a value of 1–12 in the period 1/2022–12/2022 and was intended to capture changes in 2022 relative to the level at the end of 2021.

The parameter estimates representing these pandemic-related changes are illustrated using the monthly incidence of Croatian females aged 0–17 years:

The "short-term" parameter reflects changes in the first three months of the pandemic (i.e., 3/2020–5/2020). The estimated value of this parameter is –14.630. This value implies that there was an average reduction of 14.63 per 100,000 persons in monthly incidence during the first three months of the pandemic, resulting in 14.63 × 3 = 43.89, i.e. approximately 44 fewer new patients per 100,000 persons until the end of 5/2020.

The parameter "trend21" reflects the change in trend over the period 6/2020–12/2021 and the subsequent plateau phase. The estimated value of 0.969 indicates that for each month of this period, there was an increase in incidence of 0.969 per 100,000 persons due to the pandemic relative to the previous month. Thus, in December 2021 (19th month of the period) the estimated increase in incidence attributable to the pandemic was 19 × 0.969 = 18.411 per 100,000 persons, for the whole period 6/2020–12/2021 it was

$$0.969 \times \sum_{m=1}^{19} m = 184.11$$

i.e., about 184 new patients per 100,000 persons. This is followed by the plateau phase until the end of 2022. Thus, in each month of this year, the estimated increase is the same as in December 2021, i.e., 18.411 persons. For 2022, the total increase attributable to the pandemic, as captured by trend21, is therefore 12 × 18,411 = 220,932, or approximately 221 new patients per 100 000 females aged 0–17.
Thus, over the entire period 6/2020–12/2022, the estimated increase attributable to the pandemic, as captured by trend21, is 184.11 + 220.932 = 405.042, or 405 new patients per 100 000 females.

The "trend22" parameter reflects an additional change in trend in 2022 relative to the end of 2021. The estimated value of this parameter is –0.150, which would indicate a downward trend in 2022, but this value is not statistically significant (i.e., the hypothesis that the value of this parameter is zero has not been rejected). This means that there is no sufficient evidence for a new trend in 2022 and it can be concluded that the monthly incidence remained approximately at the level of the end of 2021.

Therefore, the total increase attributable to the pandemic is the one evidenced above by the parameters short-term and trend21, i.e., -44 + 405 = 361 new patients per 100 000 females in the age 0–17 years.

Fig. S1 ARIMA model of development in antidepressants dispensing (DDD/TID) in **Croatia**

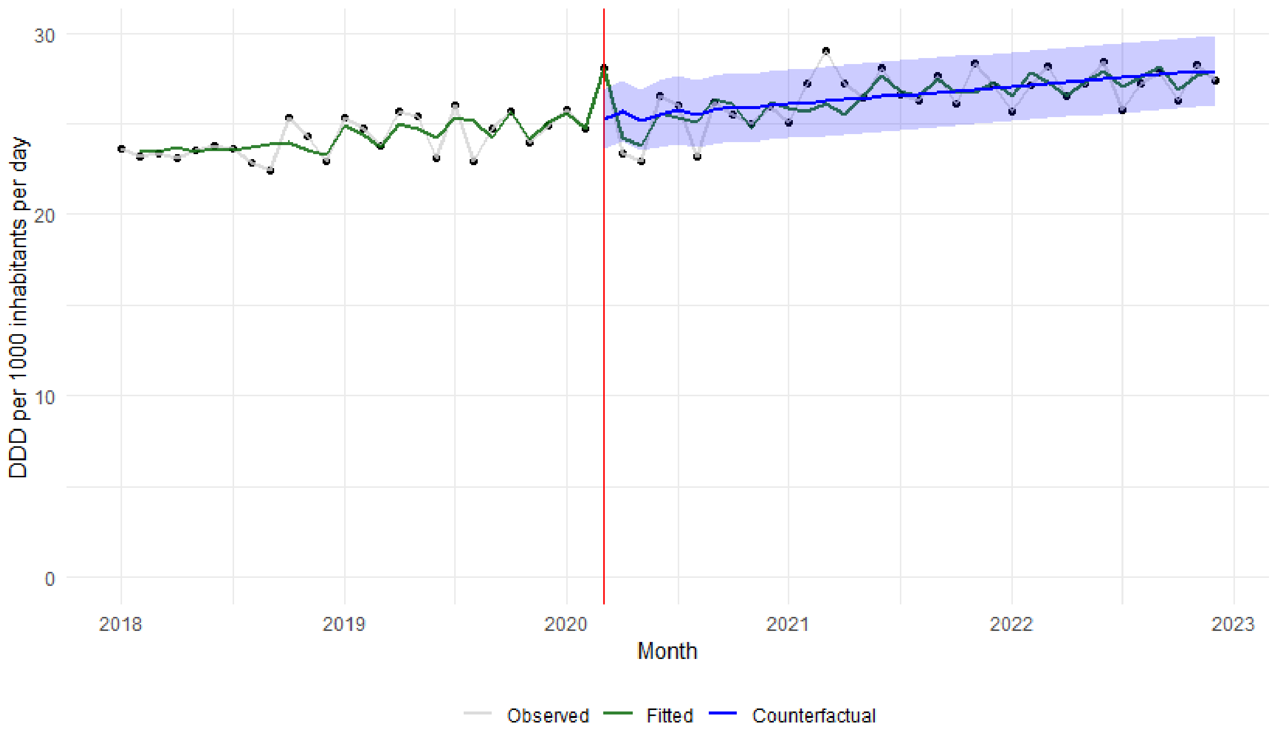

Fig. S2 ARIMA model of development in antidepressants dispensing (DDD/TID) in **Czechia**

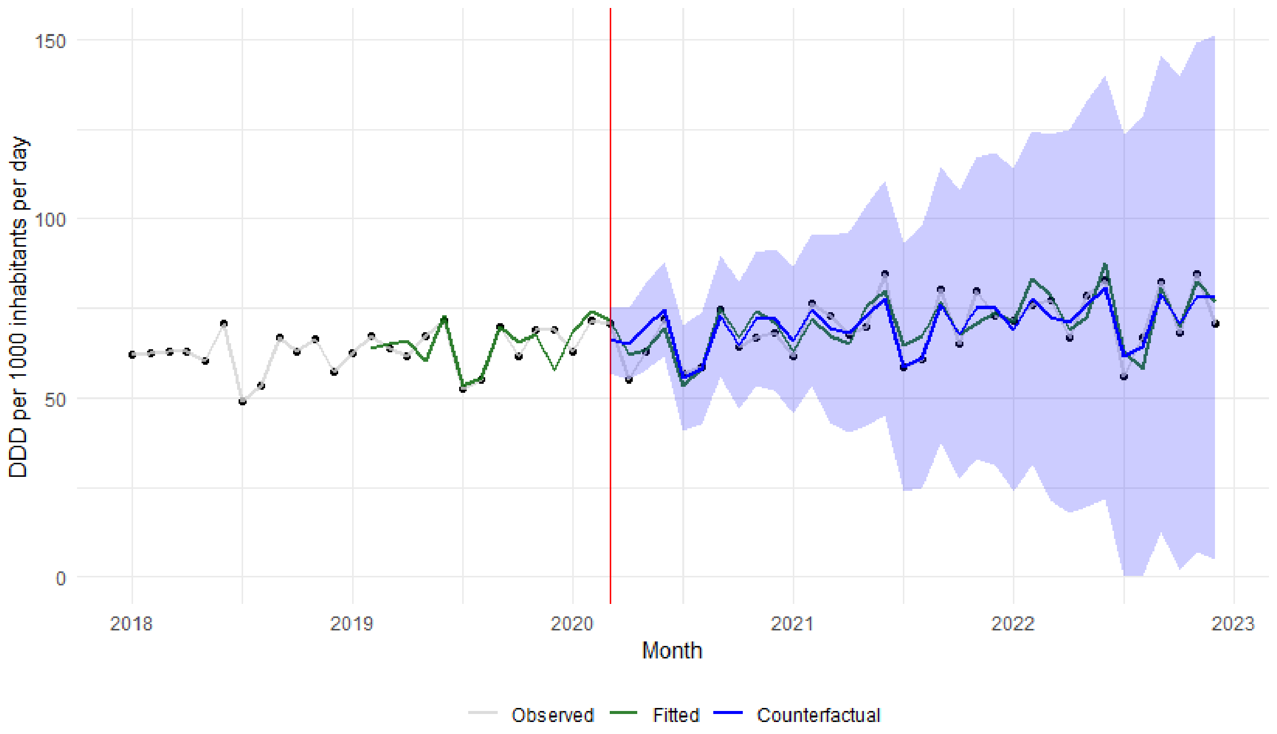

Fig. S3 ARIMA model of development in antidepressants dispensing (DDD/TID) in **England**

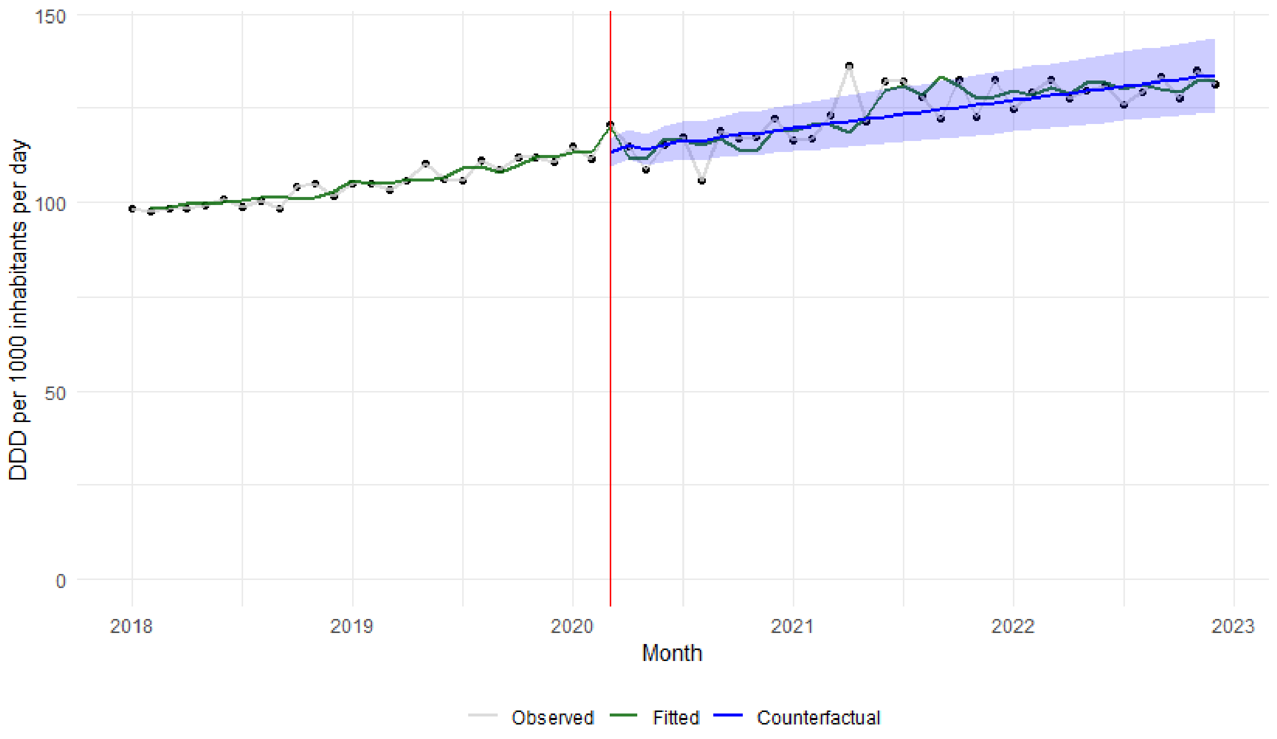

Fig. S4 ARIMA model of development in antidepressants dispensing (DDD/TID) in **Finland**

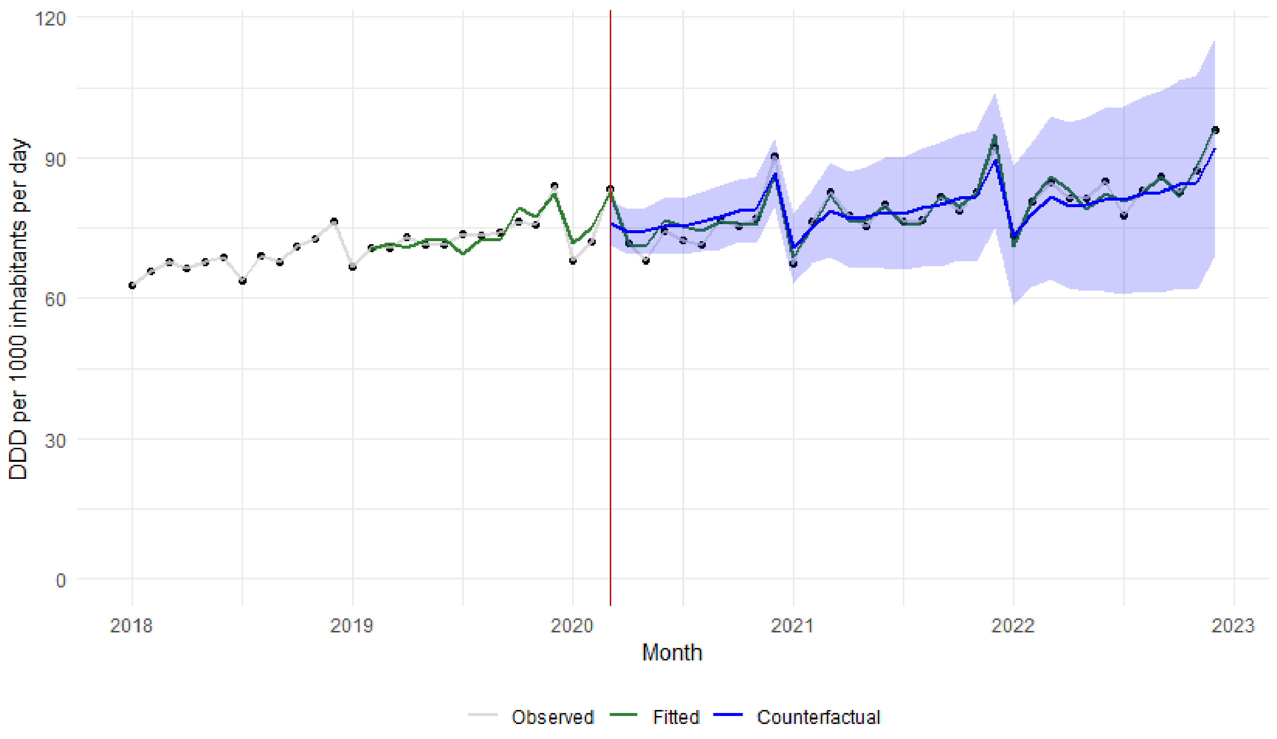

Fig. S5 ARIMA model of development in antidepressants dispensing (DDD/TID) in **Germany**

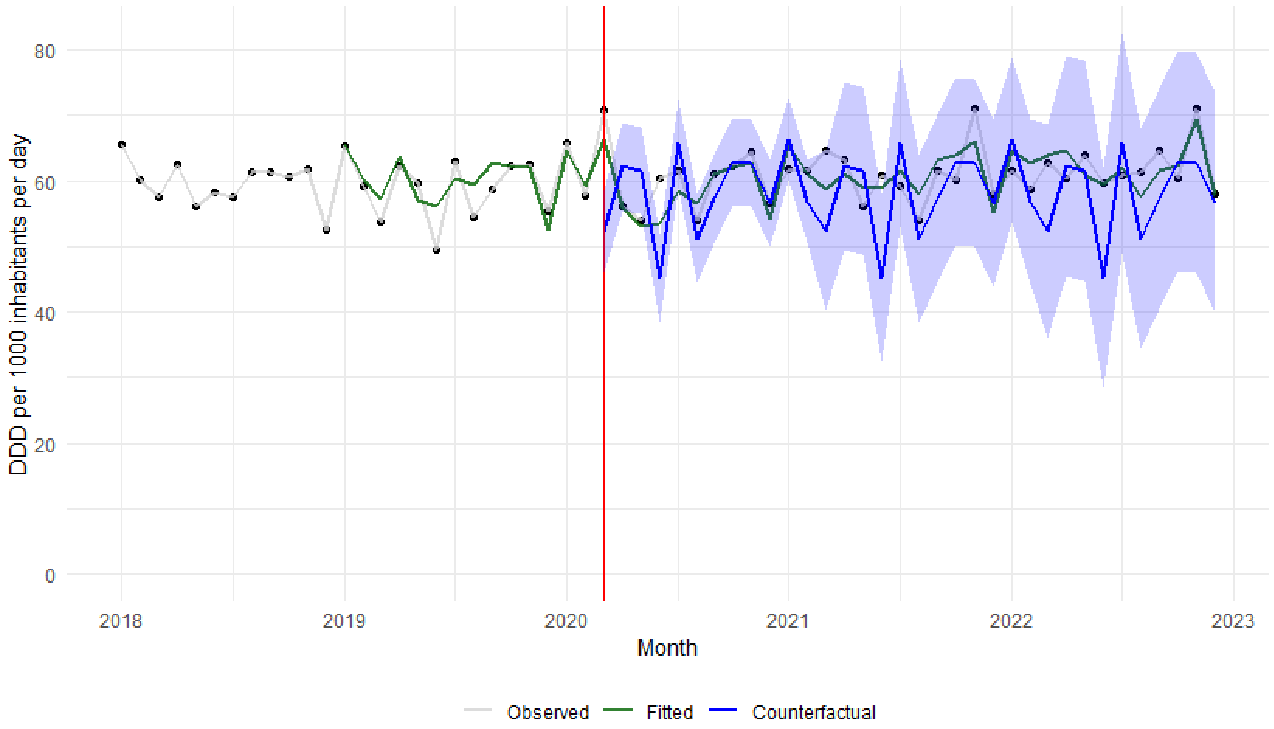

Fig. S6 ARIMA model of development in antidepressants dispensing (DDD/TID) in **Lombardy**

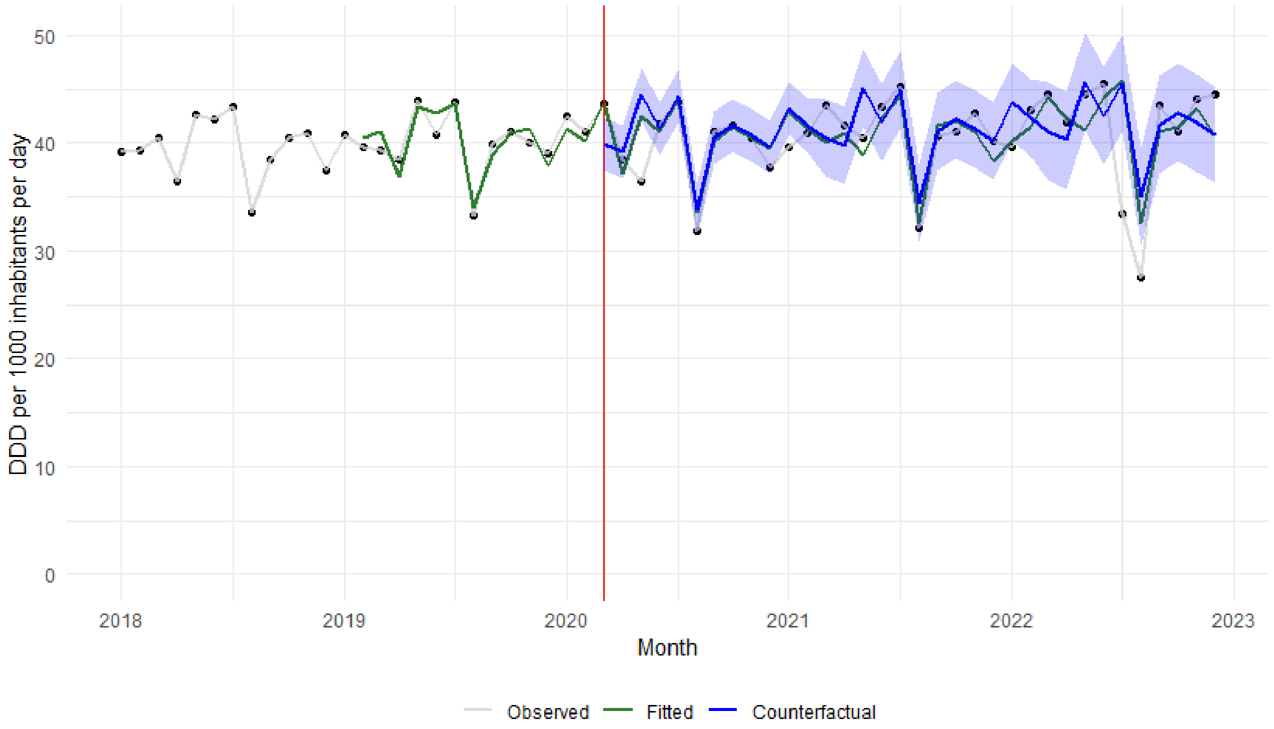

Fig. S7 ARIMA model of development in antidepressants dispensing (DDD/TID) in **Northern Irland**

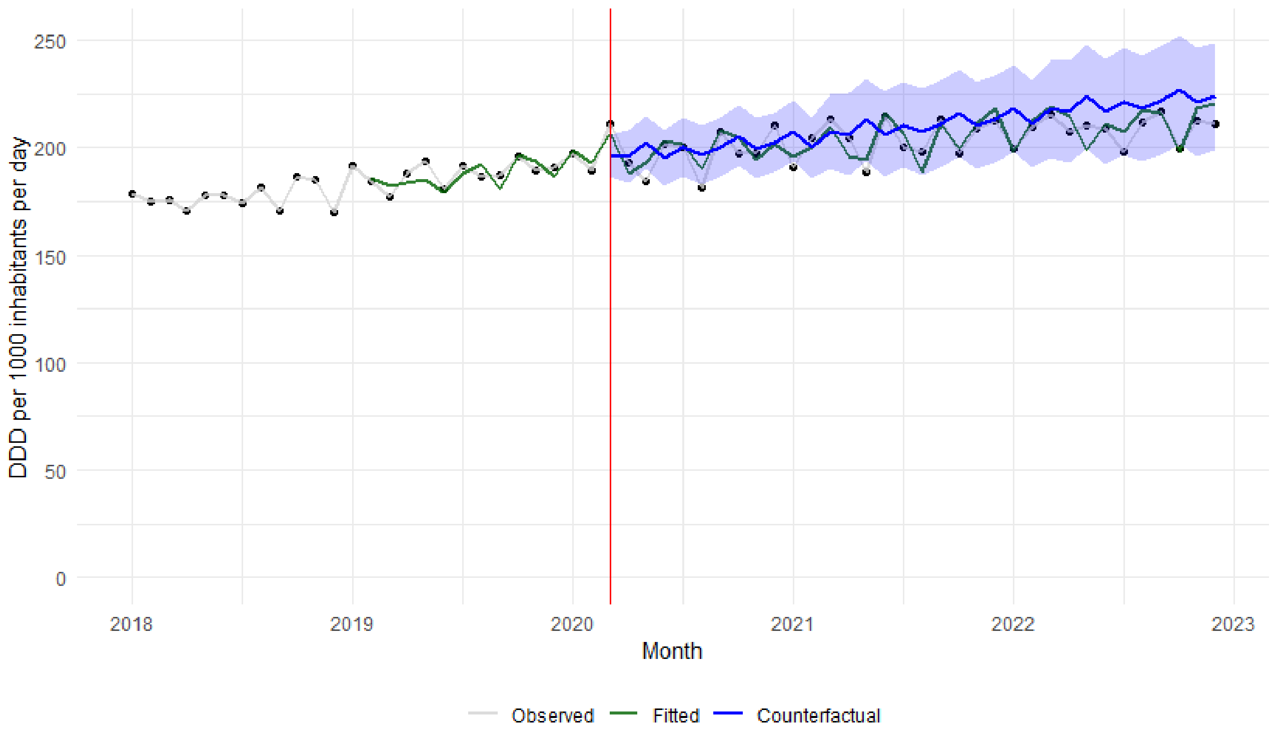

Fig. S8 ARIMA model of development in antidepressants dispensing (DDD/TID) in **Scotland**

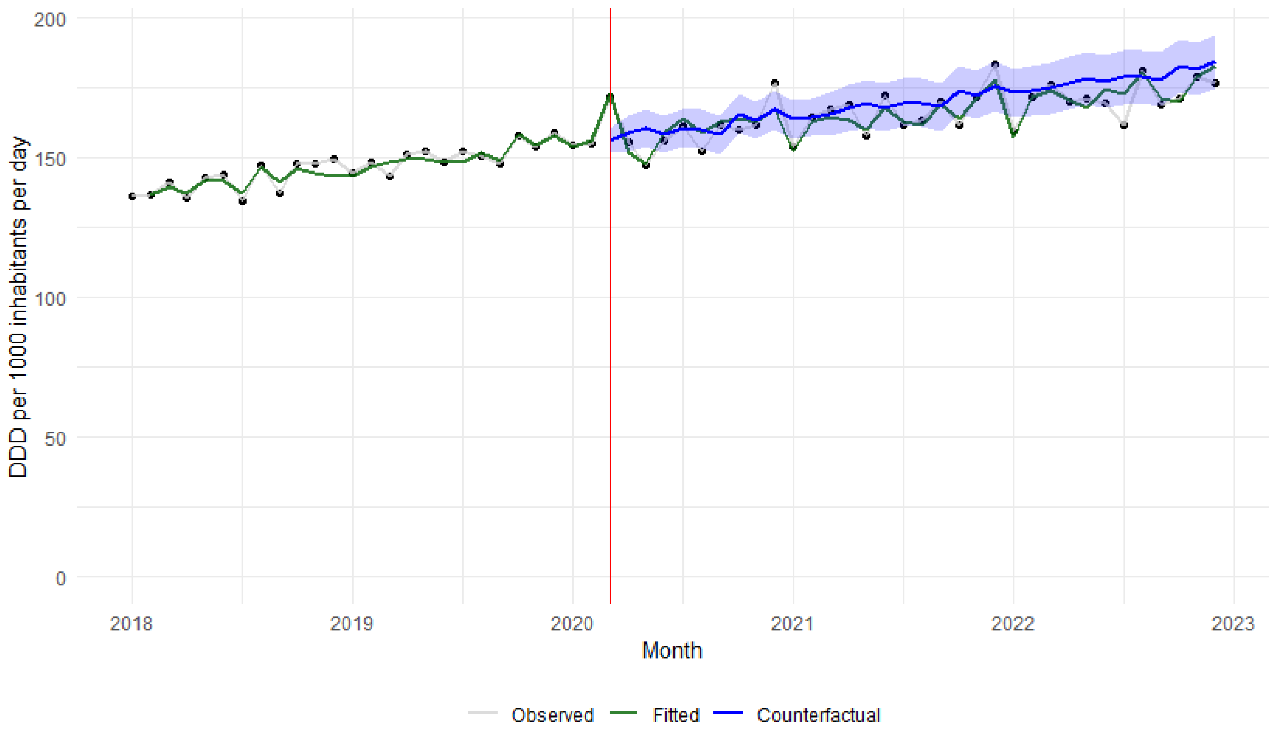

Fig. S9 ARIMA model of development in antidepressants dispensing (DDD/TID) in **Slovenia**

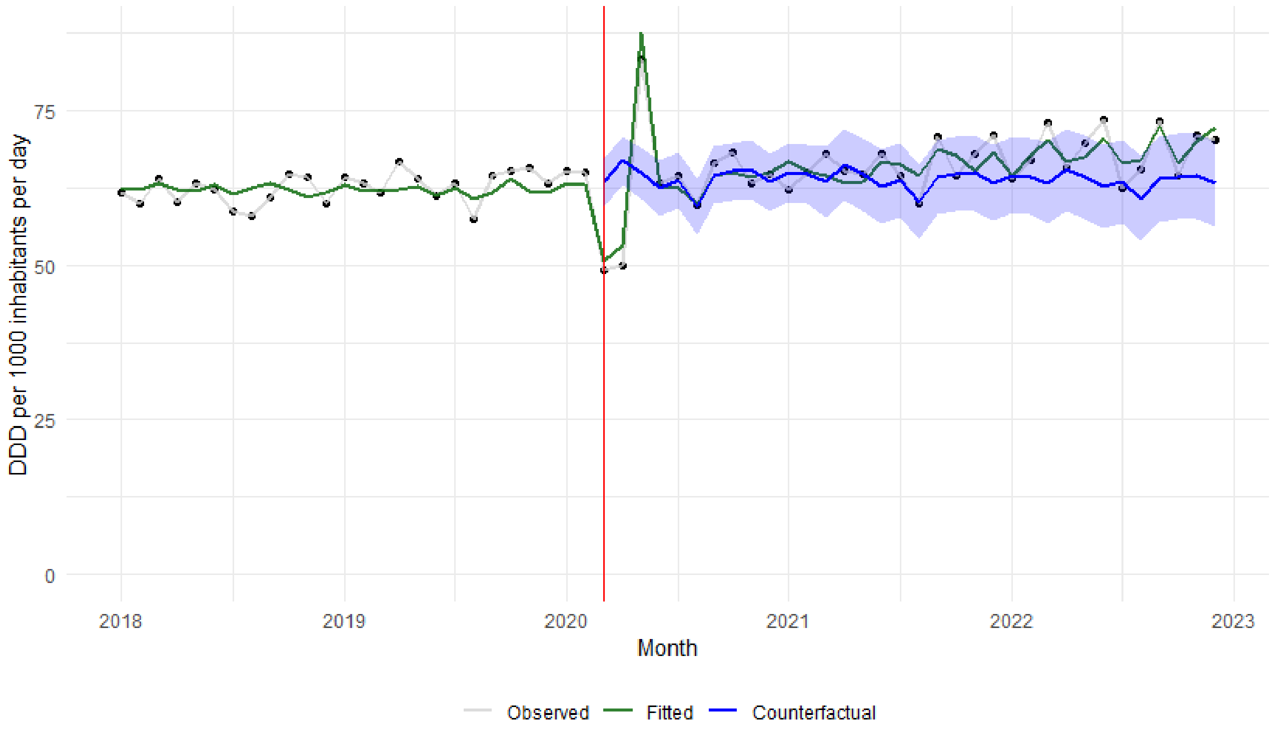

Fig. S10 ARIMA model of development in antidepressants dispensing (DDD/TID) in **Sweden**

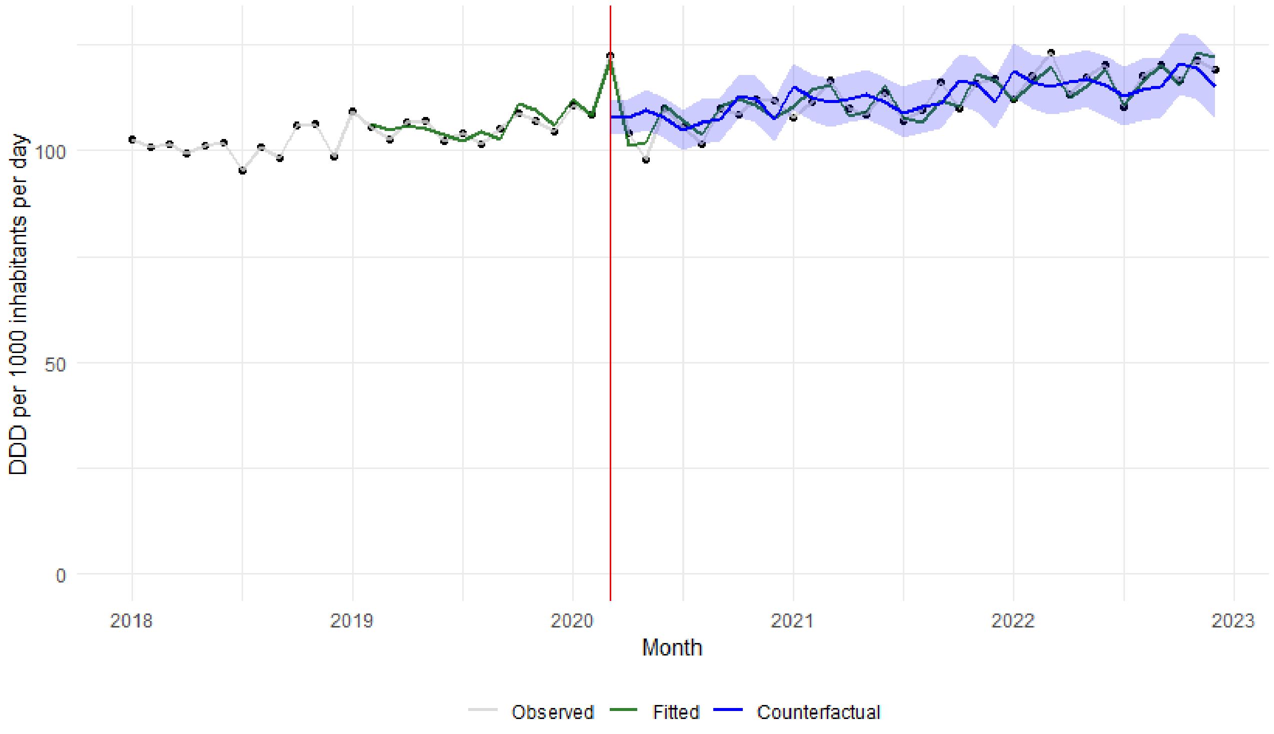

Fig. S11 ARIMA model of development in antidepressants dispensing (DDD/TID) in **Wales**

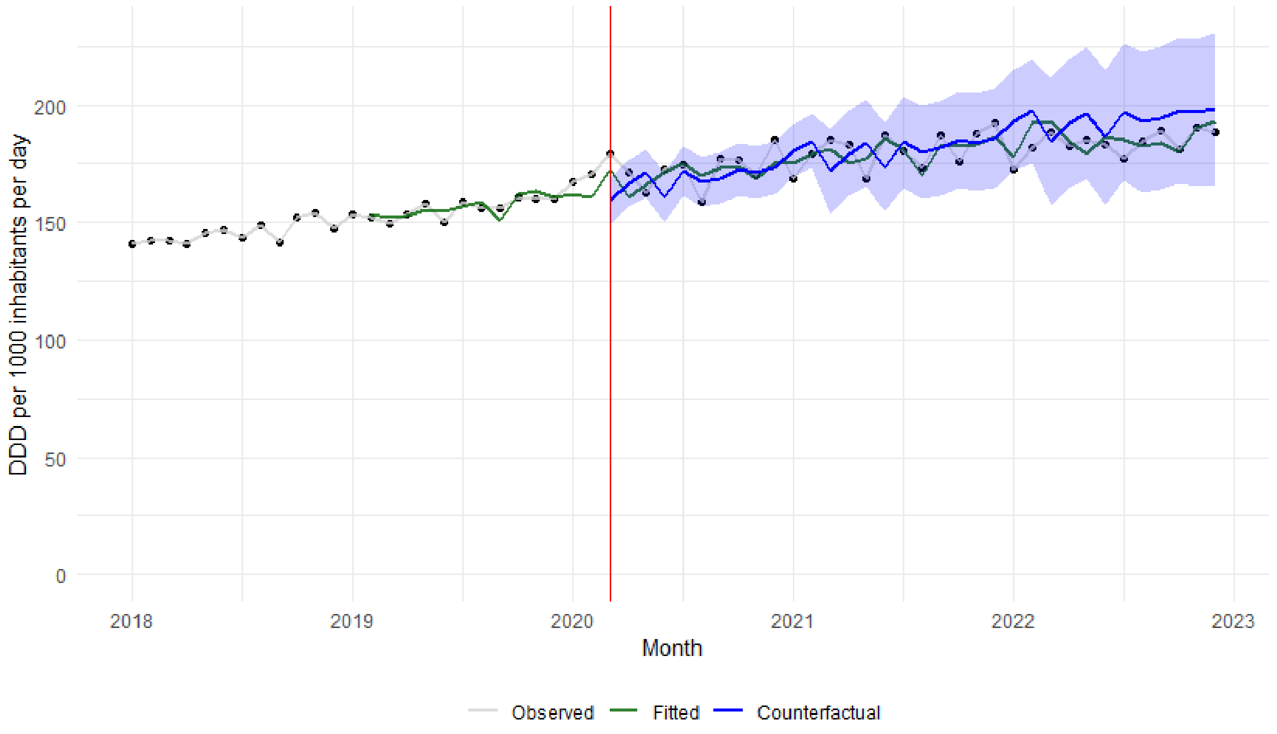

Fig. S12 Quarterly changes (%) in dispensed volumes (DDD/TID) compared to 2018, **age group 0–17** years, females (F) and males (M)

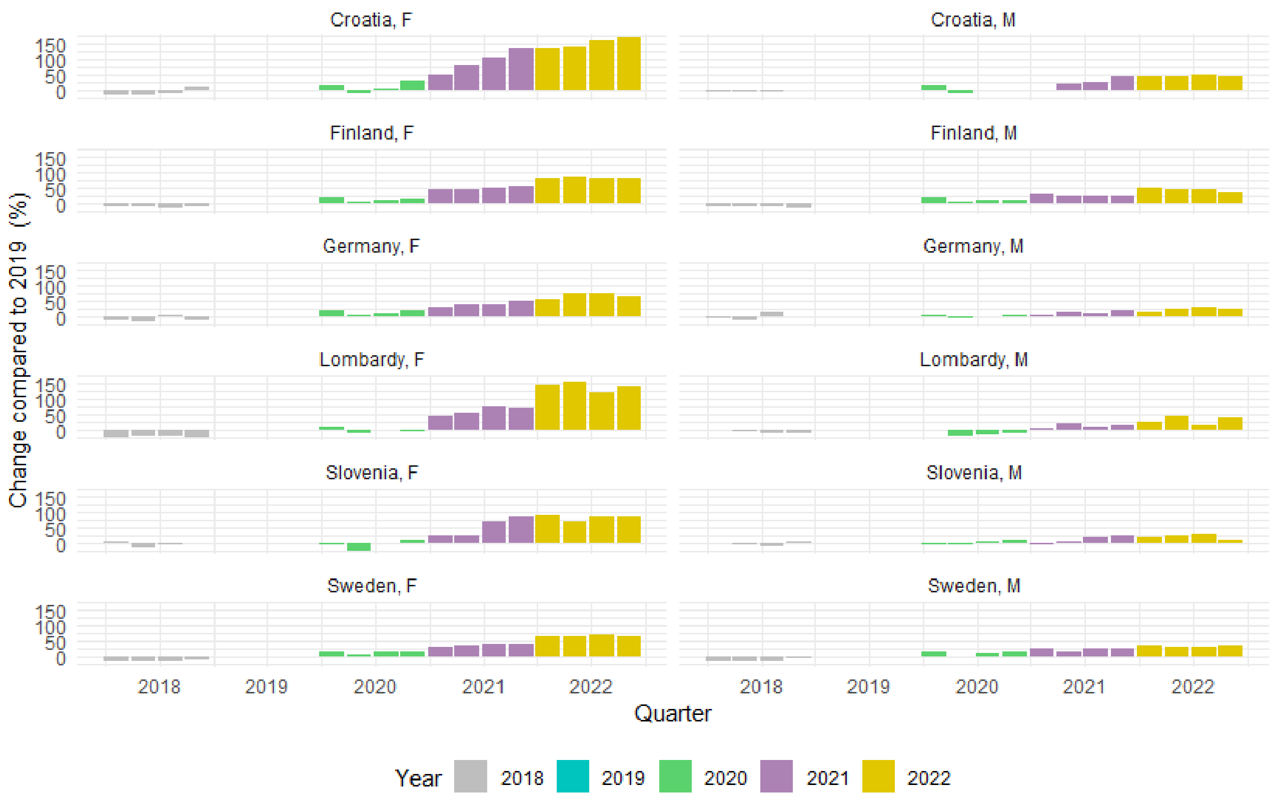

Fig. S13 Quarterly changes (%) in dispensed volumes (DDD/TID) compared to 2018, **age group 18–44** years, females (F) and males (M)

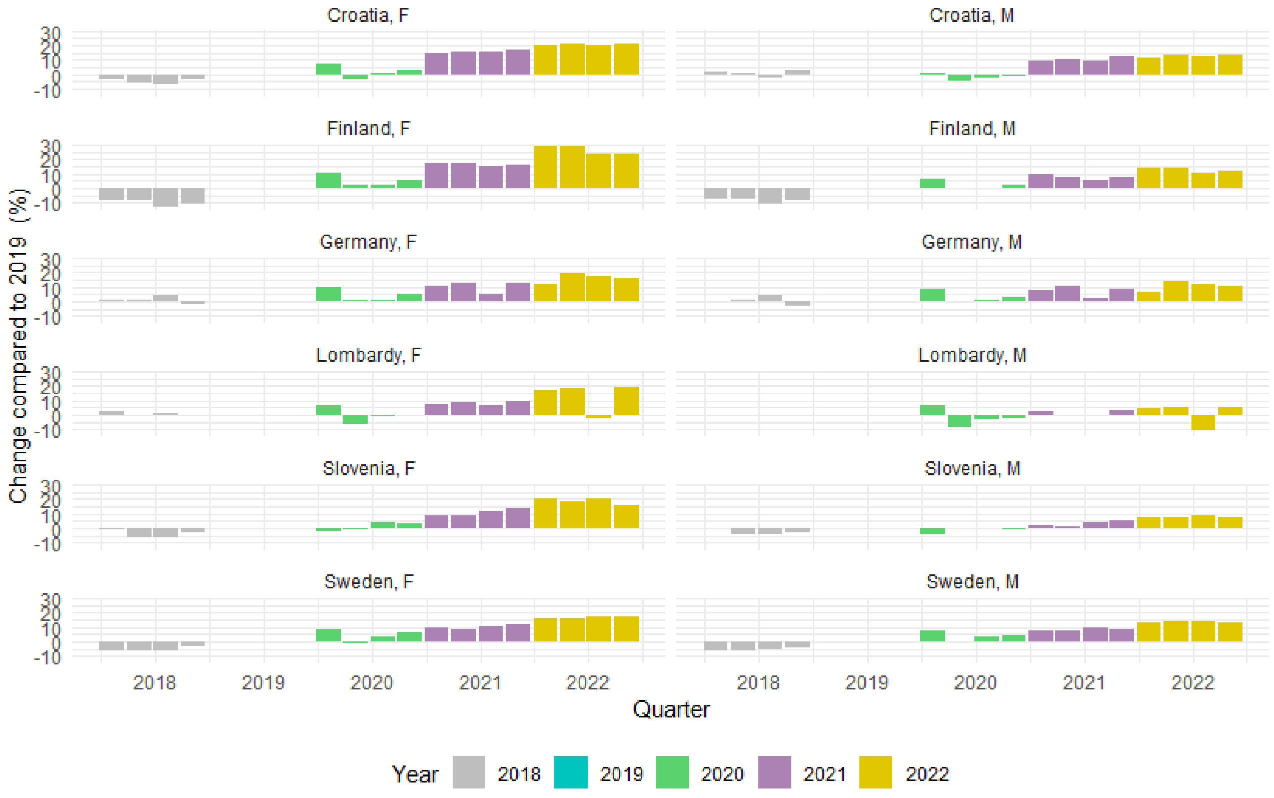

Fig. S14 Quarterly changes (%) in dispensed volumes (DDD/TID) compared to 2018, **age group 45–64** years, females (F) and males (M)

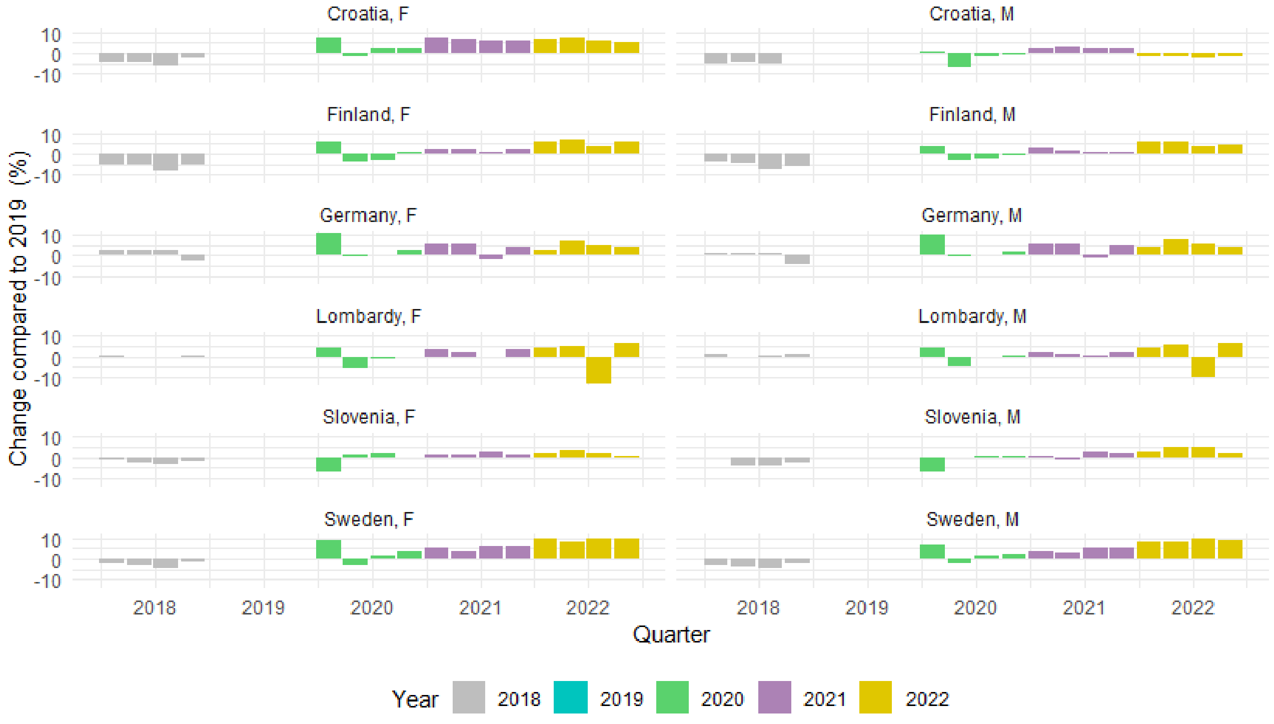

Fig. S15 Quarterly changes (%) in dispensed volumes (DDD/TID) compared to 2018, **age group 65–74** years, females (F) and males (M)

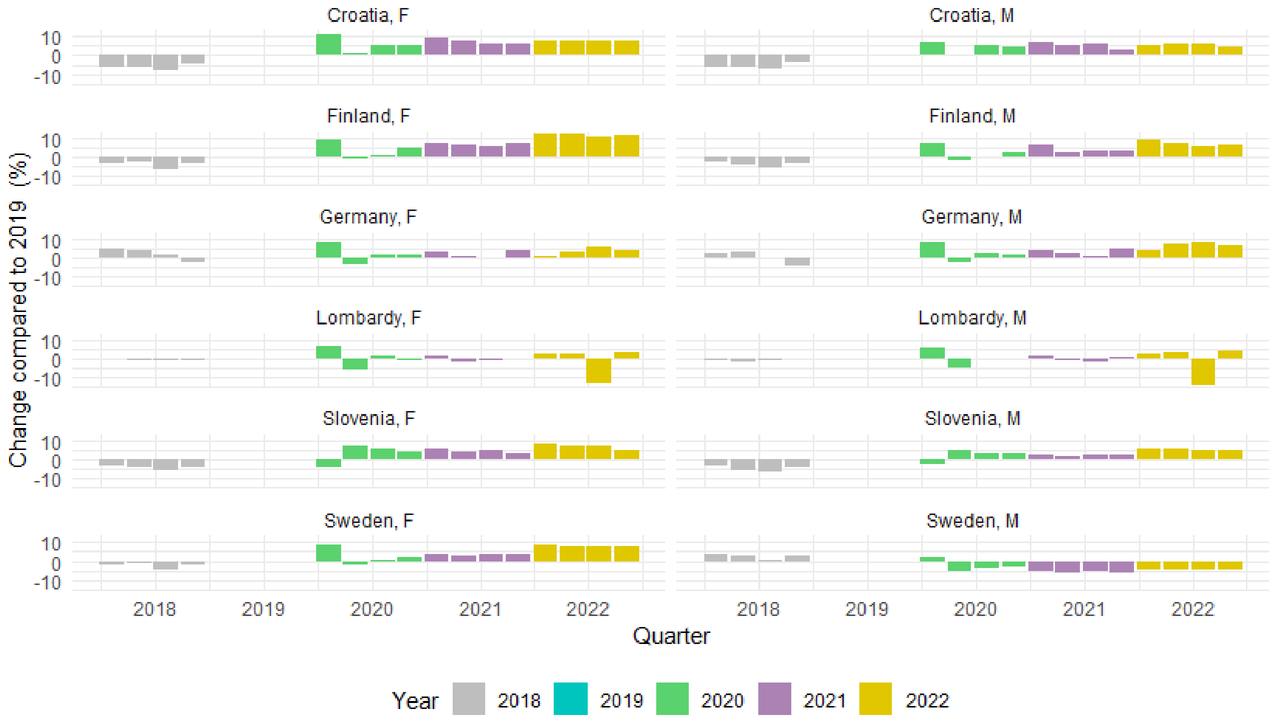

Fig. S16 Quarterly changes (%) in dispensed volumes (DDD/TID) compared to 2018, **age group 75+** years

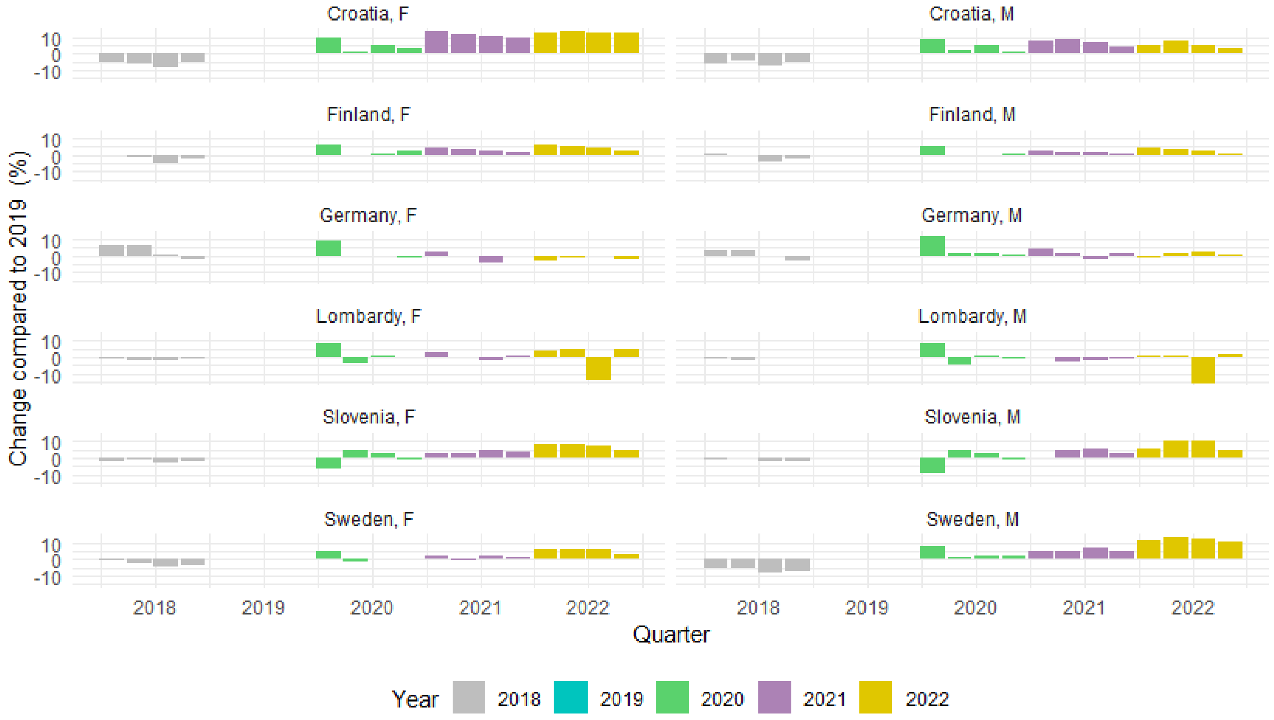

Fig. S17 Monthly incidence in the **age group 0–17** years for females (F) and males (M)

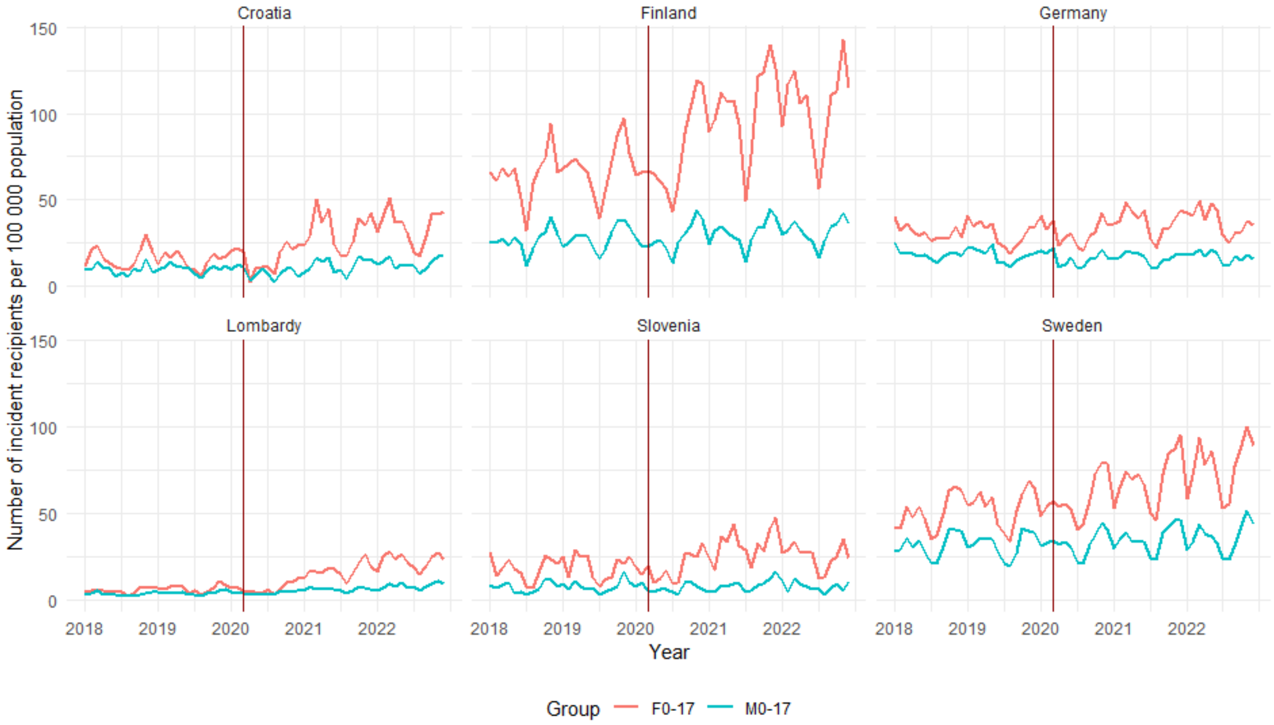

Fig. S18 Monthly incidence in the **age group 18–44** years for females (F) and males (M)

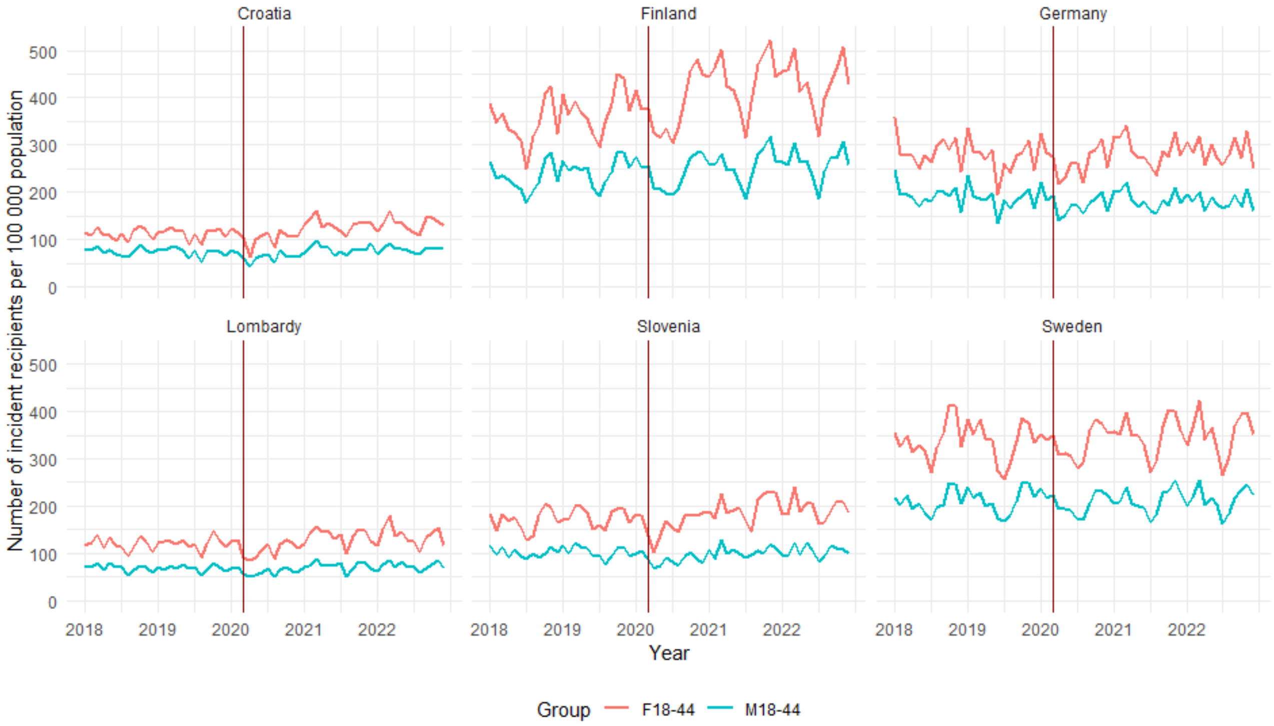

Fig. S19 Monthly incidence in the **age group 45–64** years for females (F) and males (M)

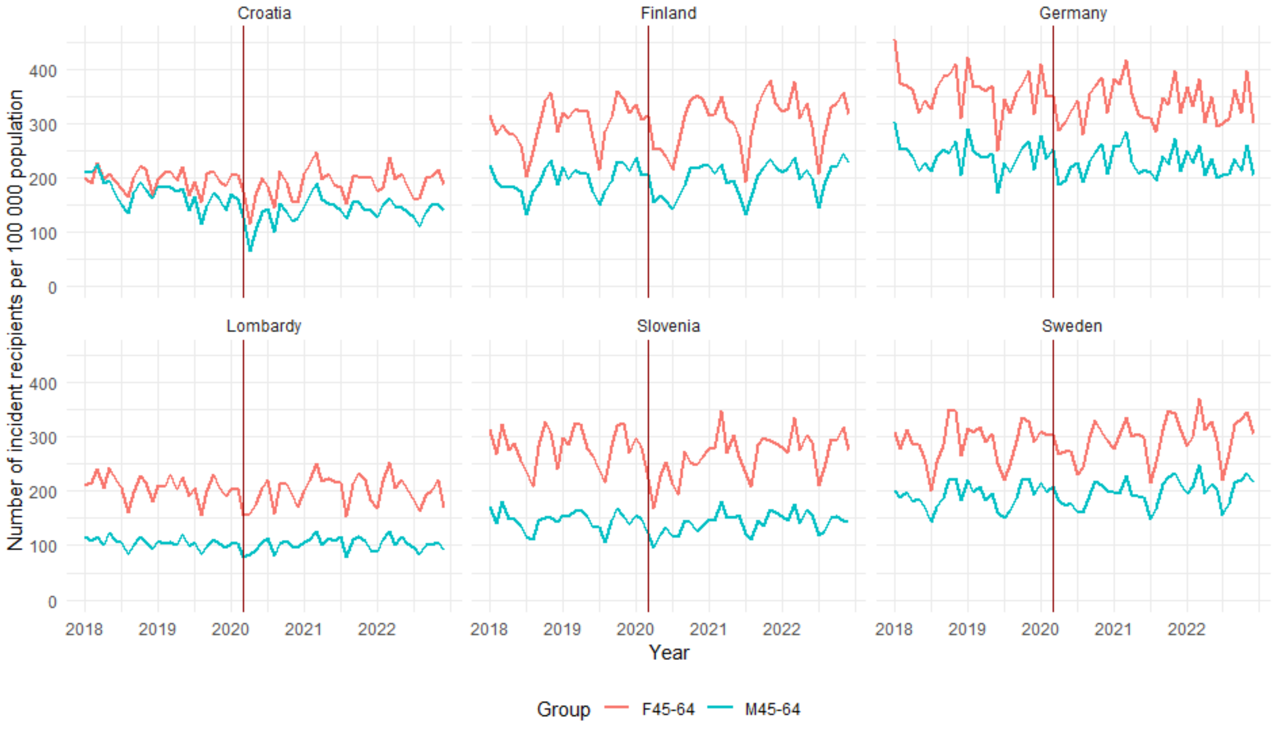

Fig. S20 Monthly incidence in the **age group 65–74** years for females (F) and males (M)

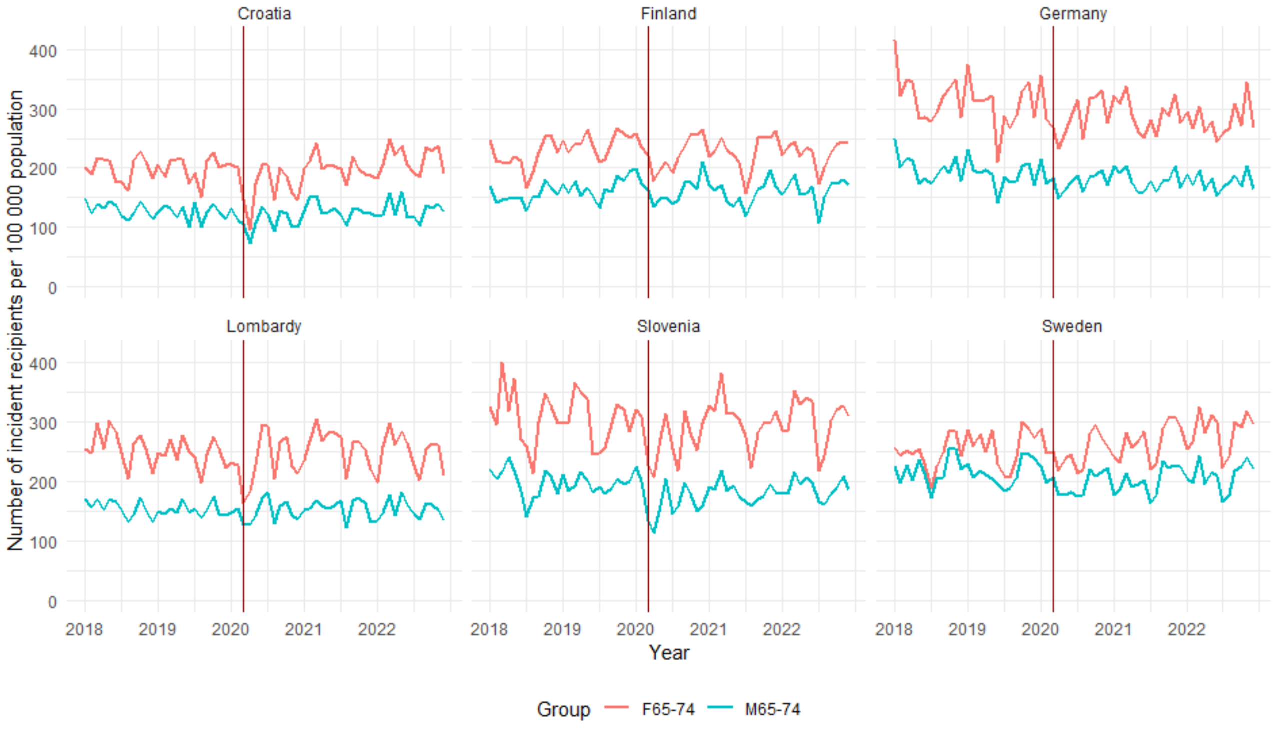

Fig. S21: Monthly incidence in the **age group 75+** years for females (F) and males (M)

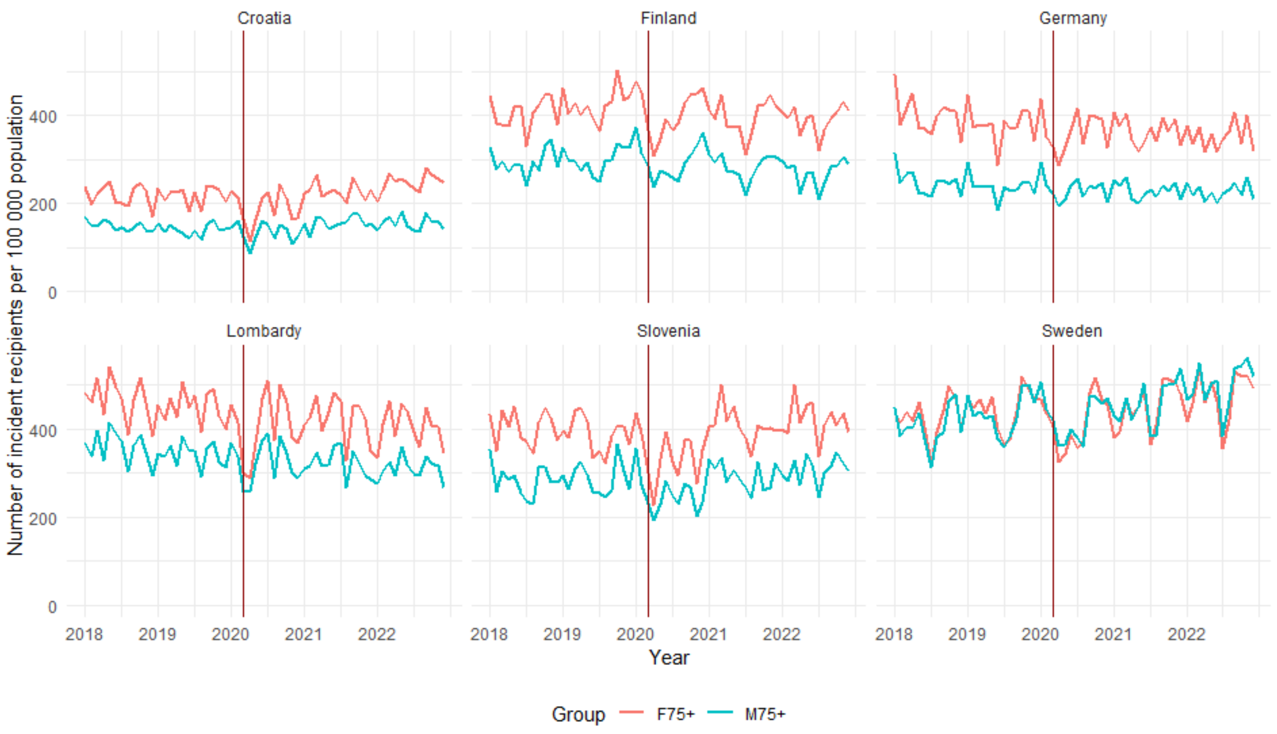

Fig. S22 Quarterly changes (%) in incidence per 100 000 for 2019–2022 relative to 2018 in **females** in the **age group 0–17** years

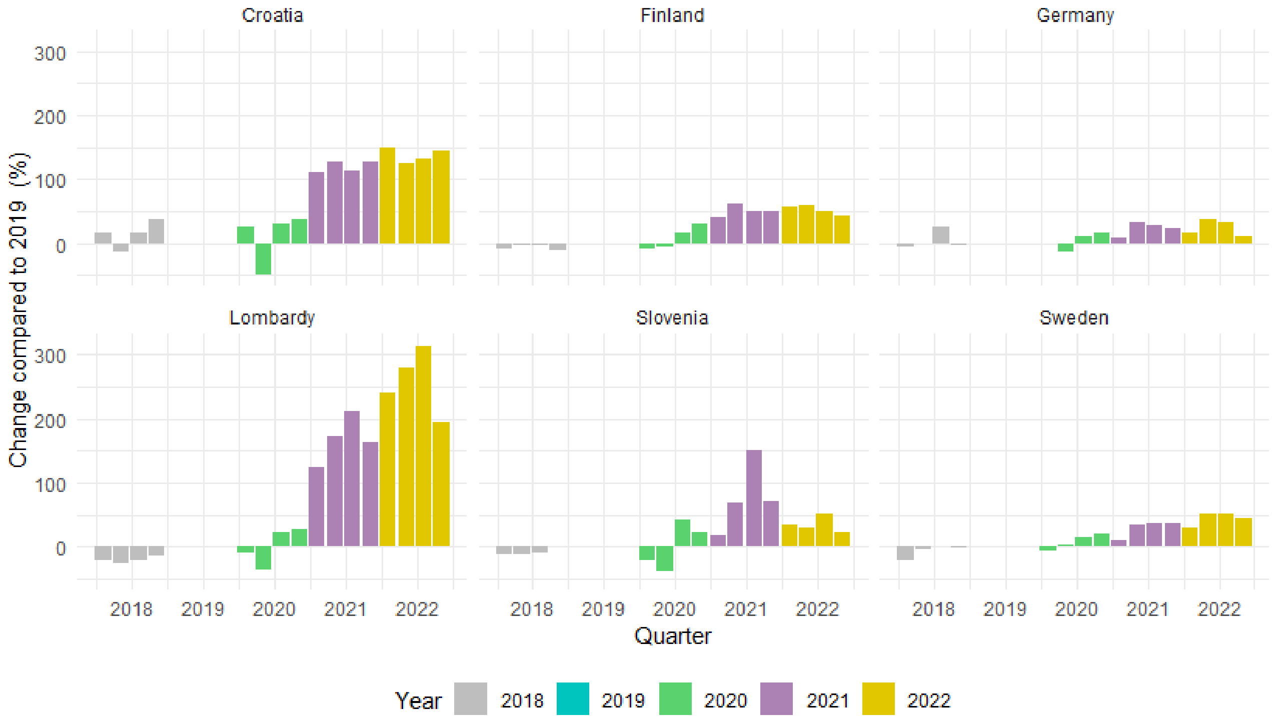

Fig. S23 Quarterly changes (%) in incidence per 100 000 for 2019–2022 relative to 2018 in **males** in the **age group 0–17** years

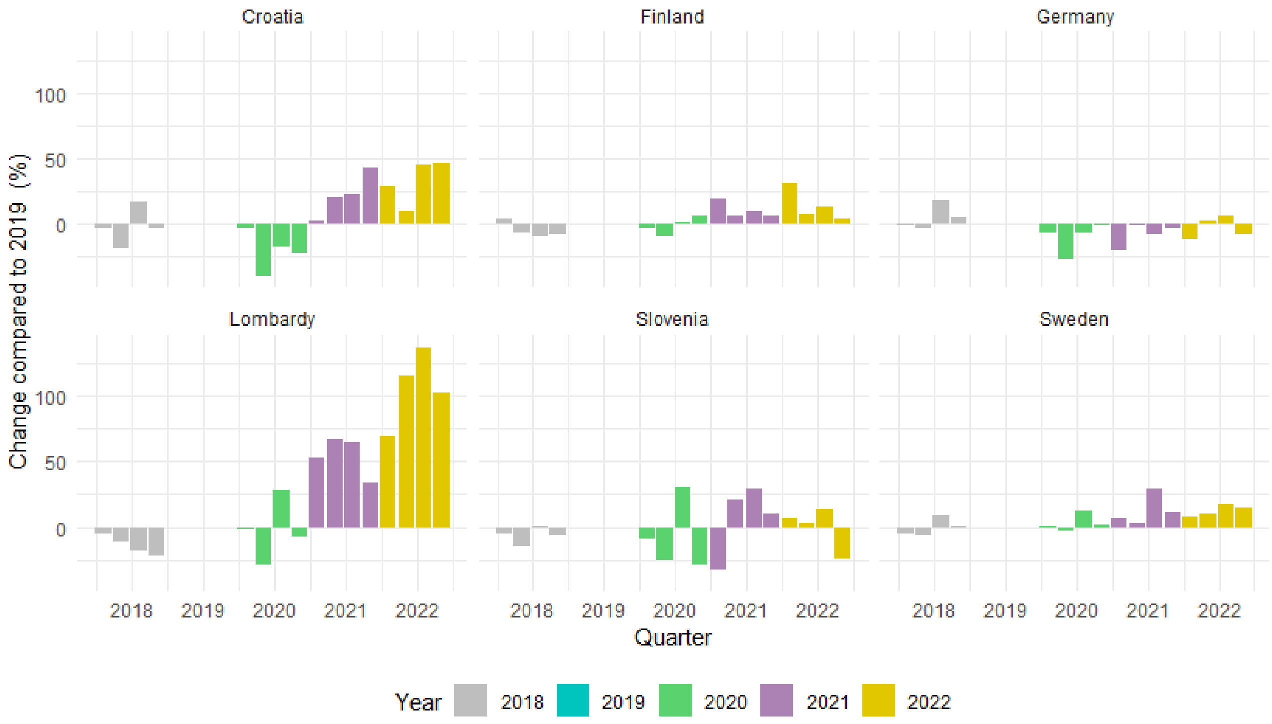

Fig. S24 Quarterly changes (%) in incidence per 100 000 for 2019–2022 relative to 2018 in **females** in the **age group 18–44** years

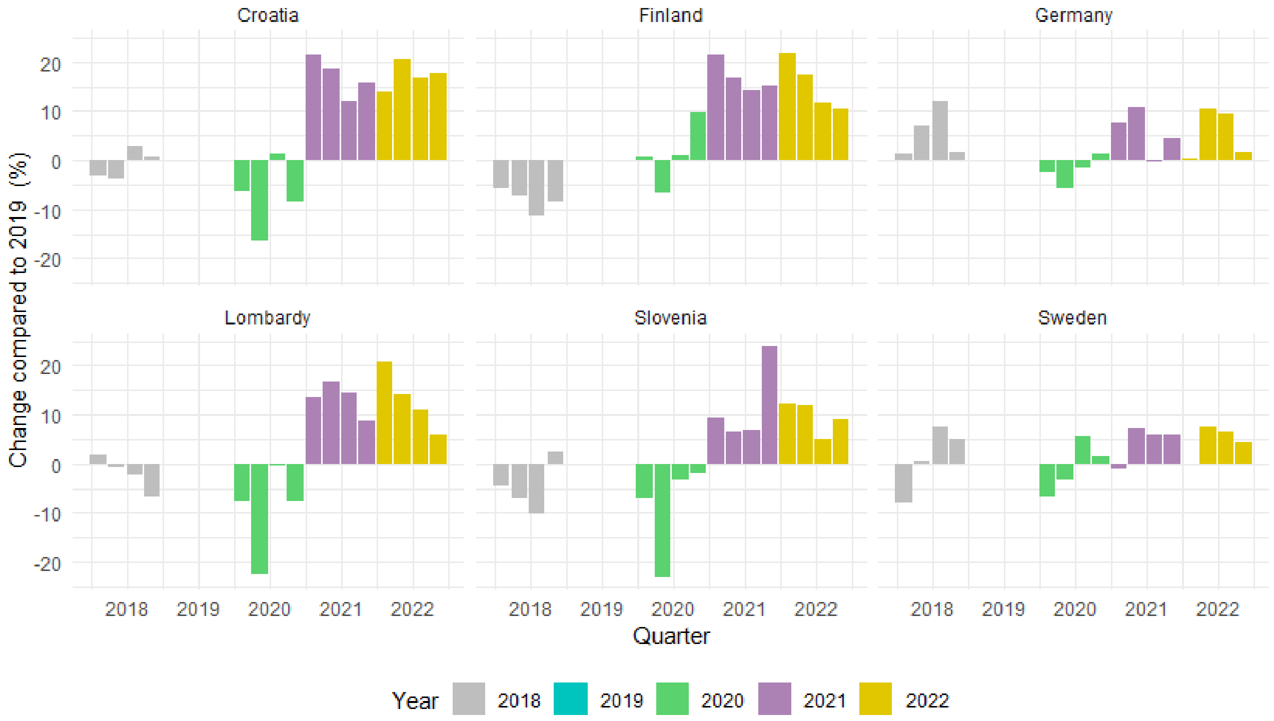

Fig. S25 Quarterly changes (%) in incidence per 100 000 for 2019–2022 relative to 2018 in **males** in the **age group 18–44** years

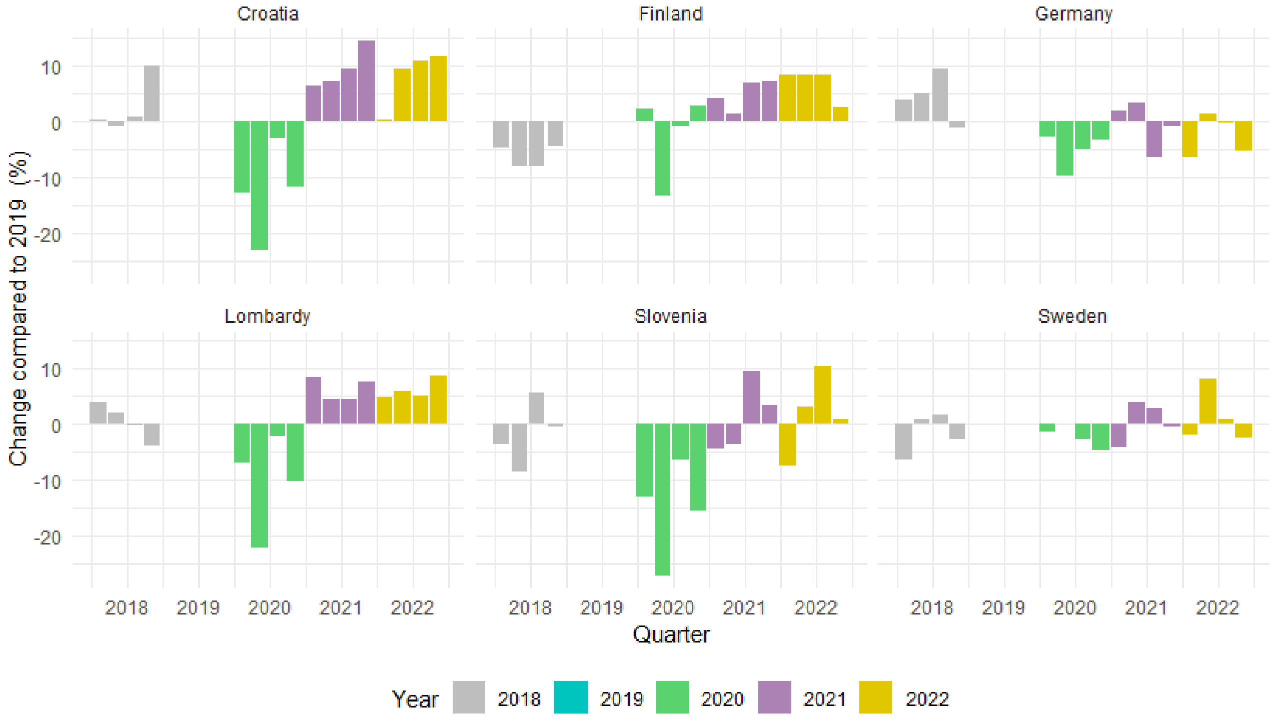

Fig. S26 Quarterly changes (%) in incidence per 100 000 for 2019–2022 relative to 2018 in **females** in the **age group 45–64** years

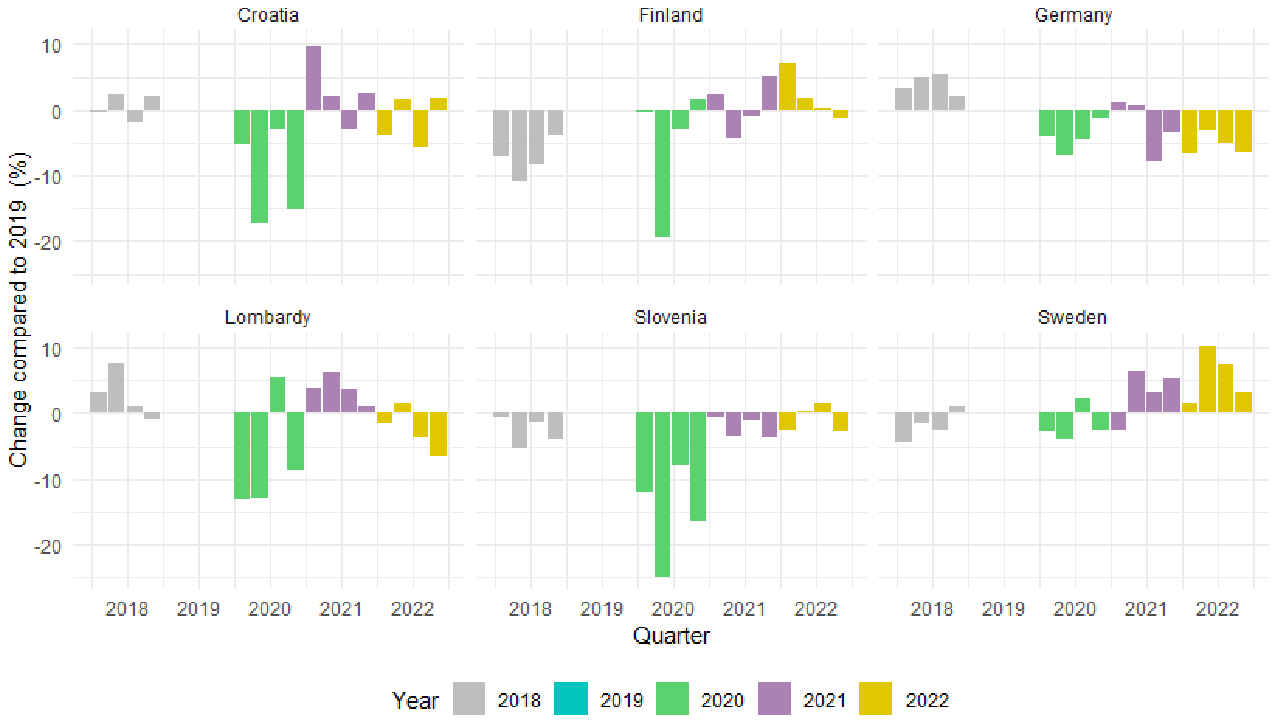

Fig. S27 Quarterly changes (%) in incidence per 100 000 for 2019–2022 relative to 2018 in **males** in the **age group 45–64** years

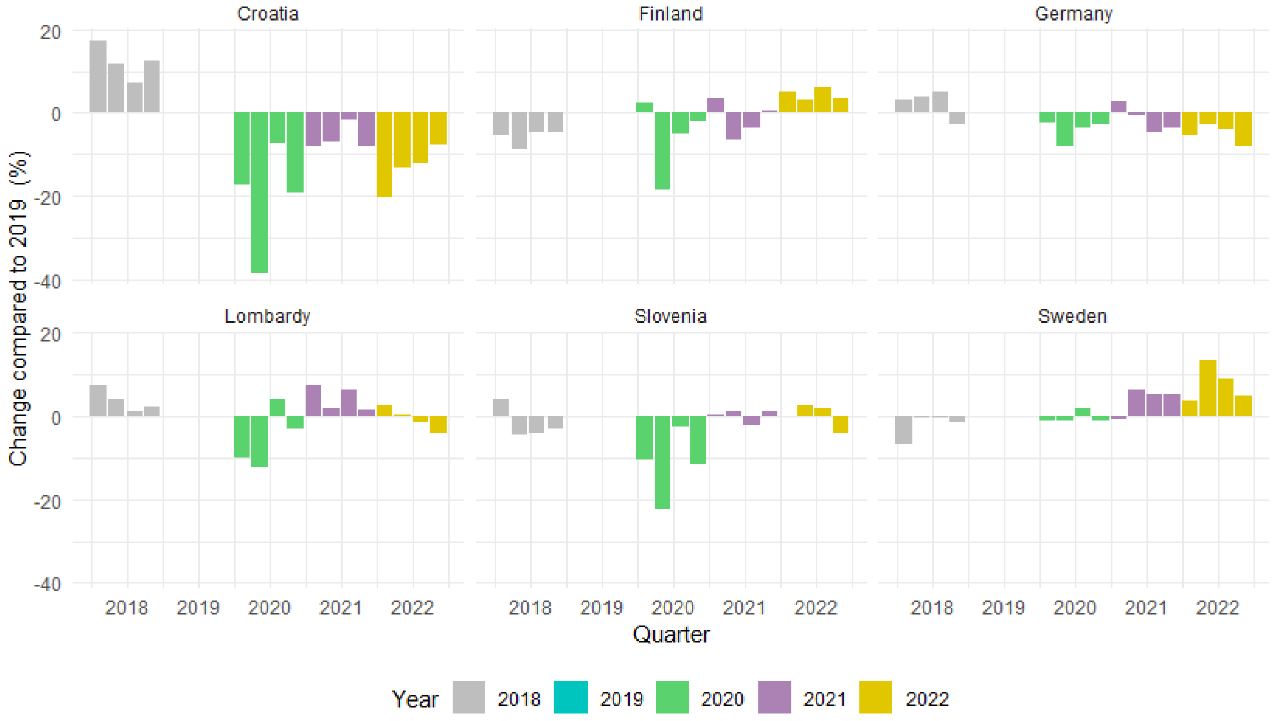

Fig. S28 Quarterly changes (%) in incidence per 100 000 for 2019–2022 relative to 2018 in **females** in the **age group 65–74** years

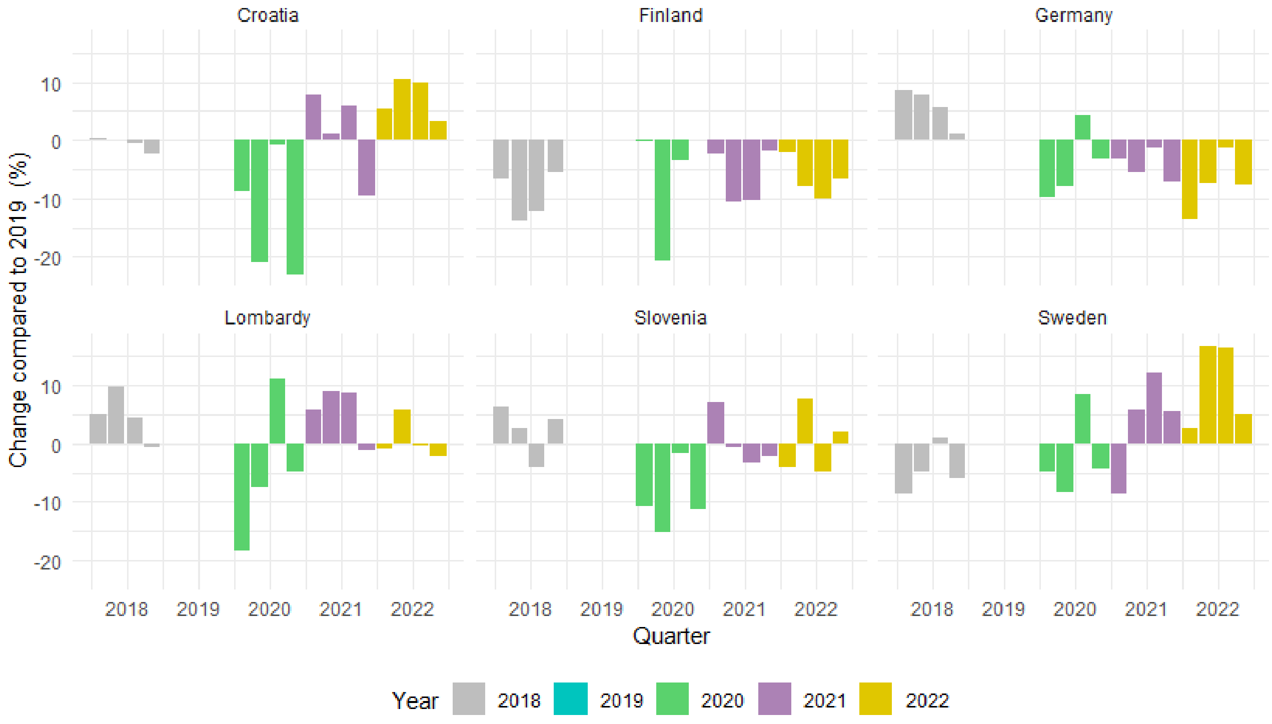

Fig. S29 Quarterly changes (%) in incidence per 100 000 for 2019–2022 relative to 2018 in **males** in the **age group 65–74** years

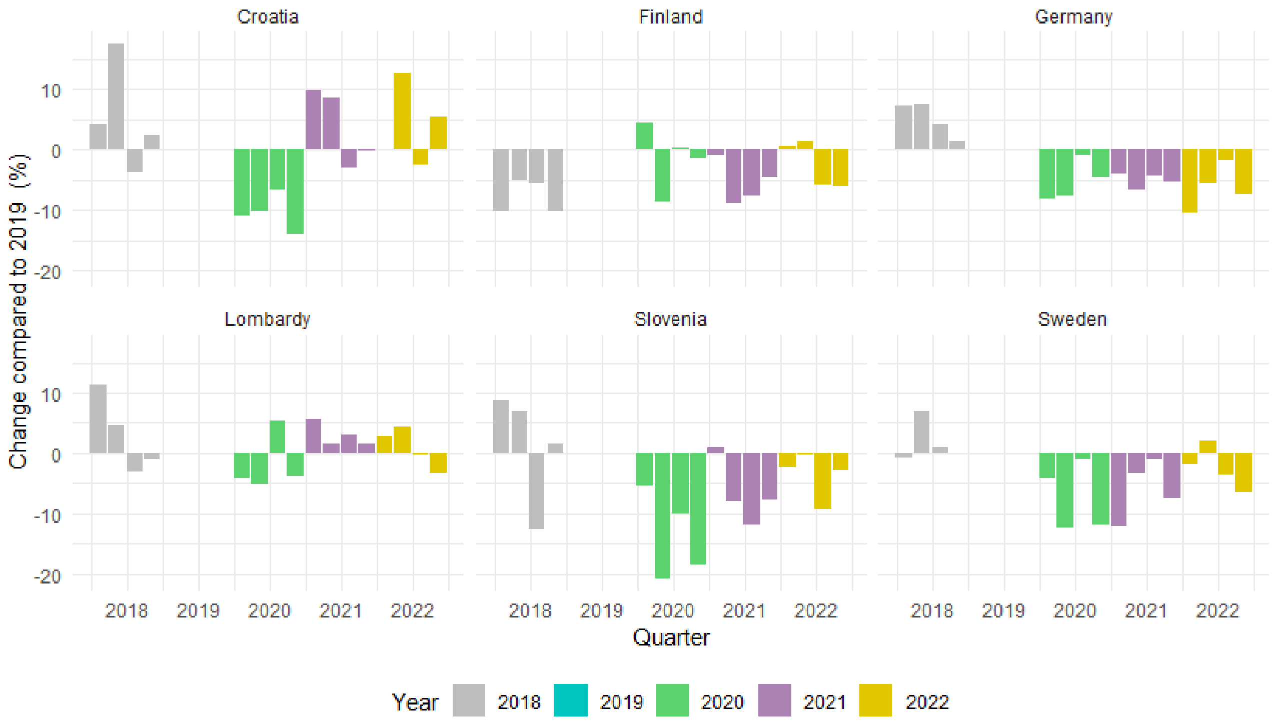

Fig. S30 Quarterly changes (%) in incidence per 100 000 for 2019–2022 relative to 2018 in **females** in the **age group 75+** years

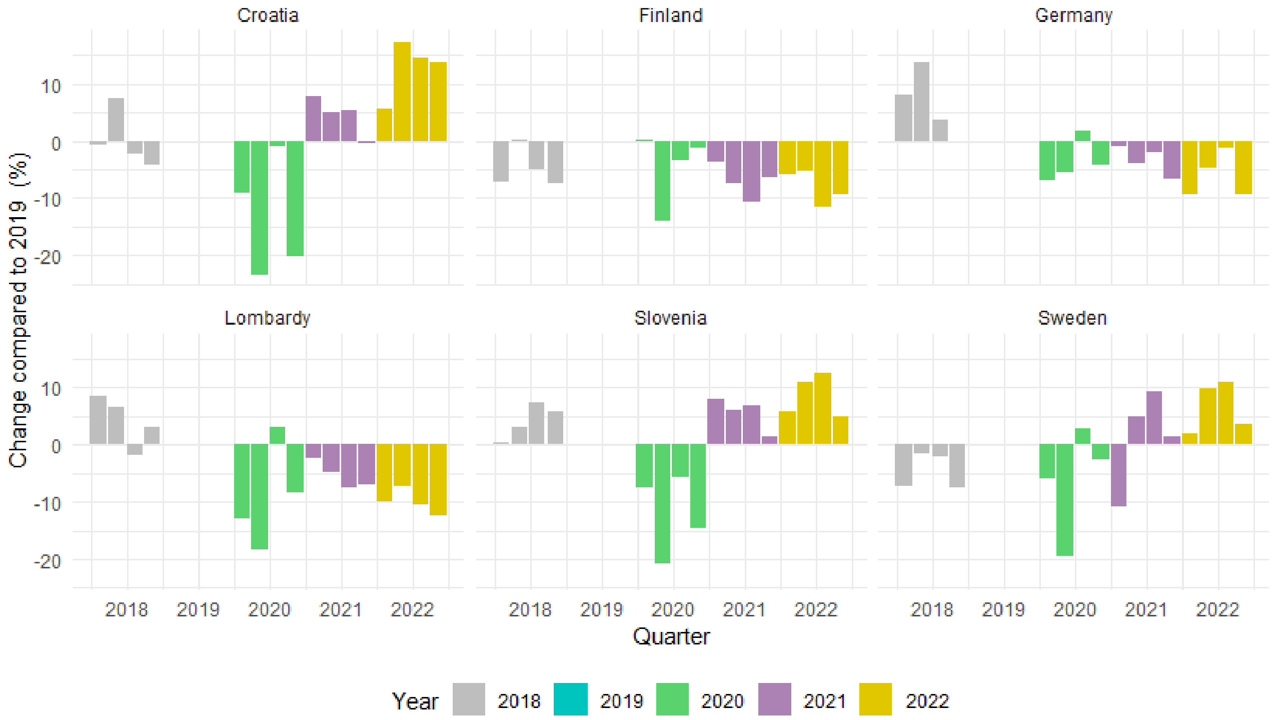

Fig. S31 Quarterly changes (%) in incidence per 100 000 for 2019–2022 relative to 2018 in **males** in the **age group 75+** years

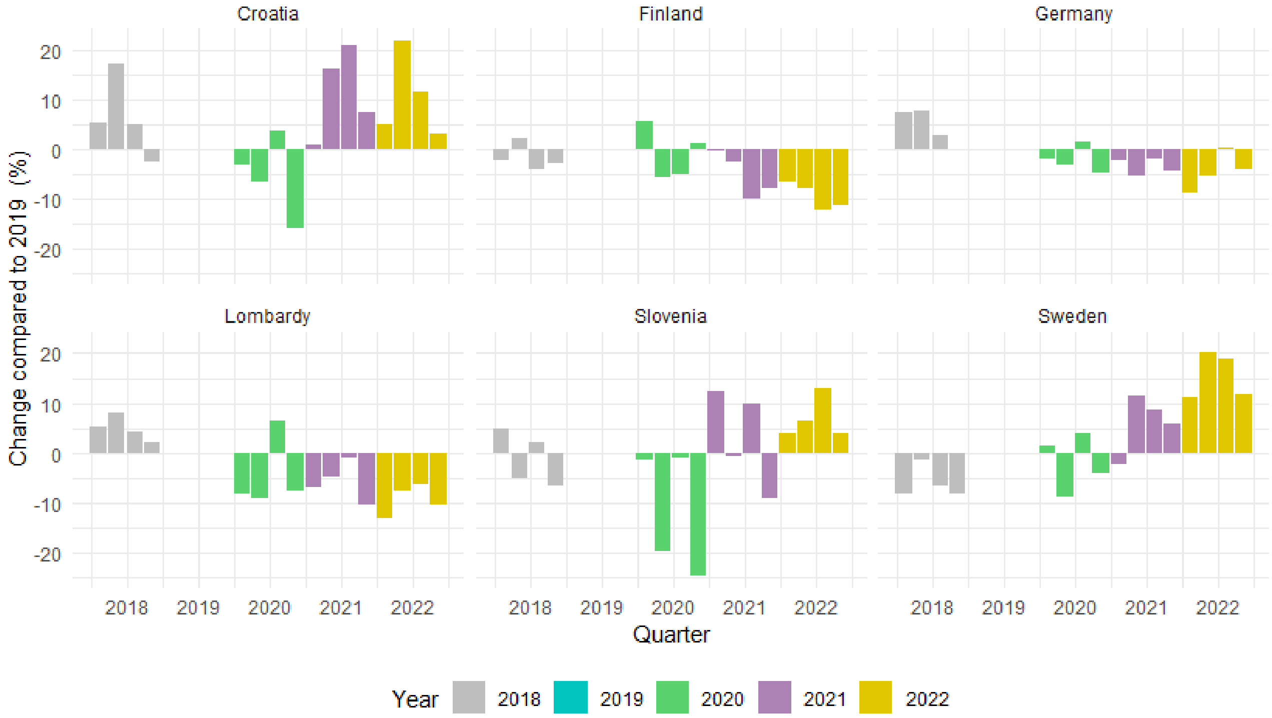

Fig. S32 ARIMA model of development in monthly incidence (per 100 000) in **females** in the age group 0–17 years, **Croatia**

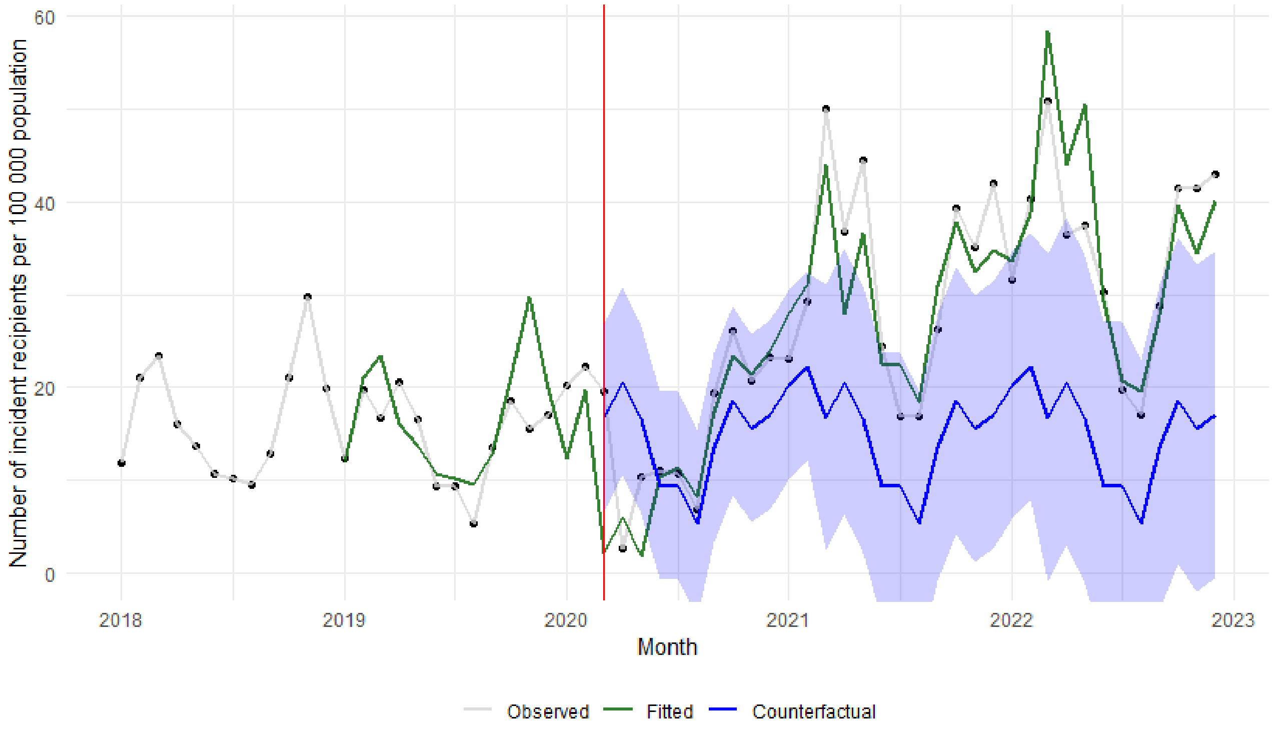

Fig. S33 ARIMA model of development in monthly incidence (per 100 000) in **males** in the age group 0–17 years, **Croatia**

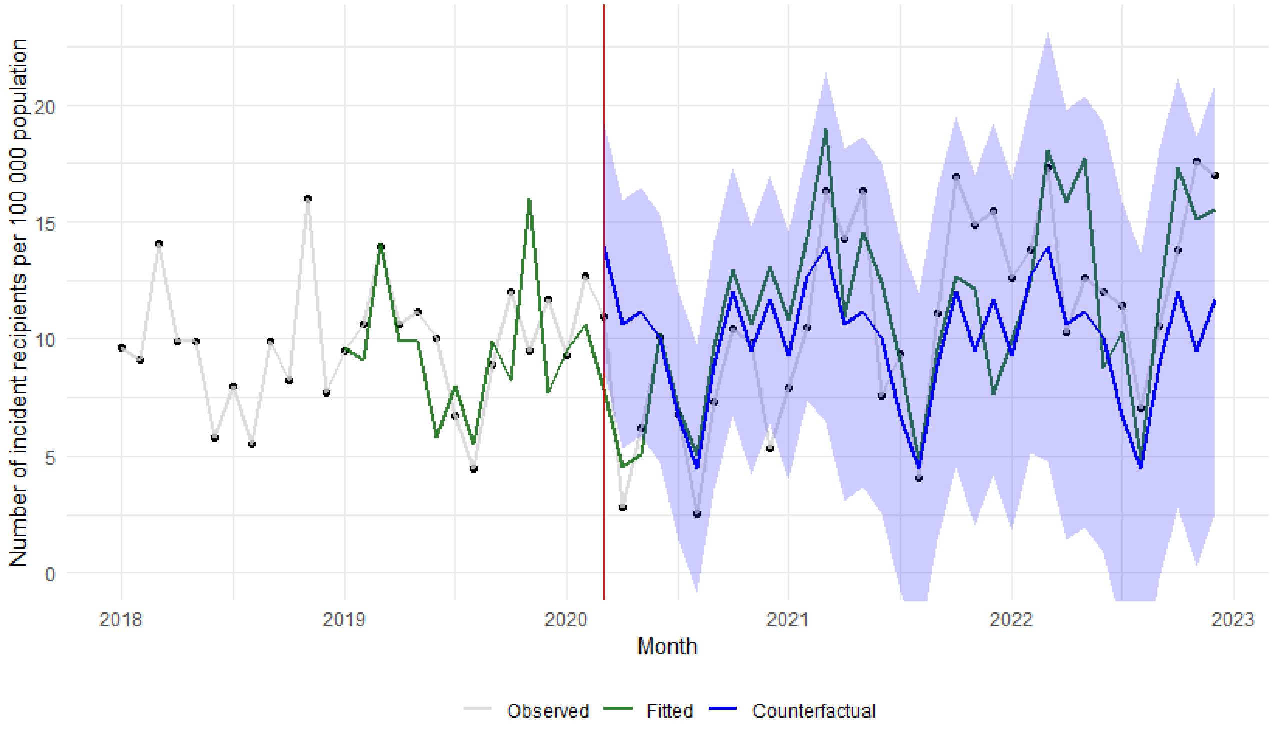

Fig. S34 ARIMA model of development in monthly incidence (per 100 000) in **females** in the age group 0–17 years, **Finland**

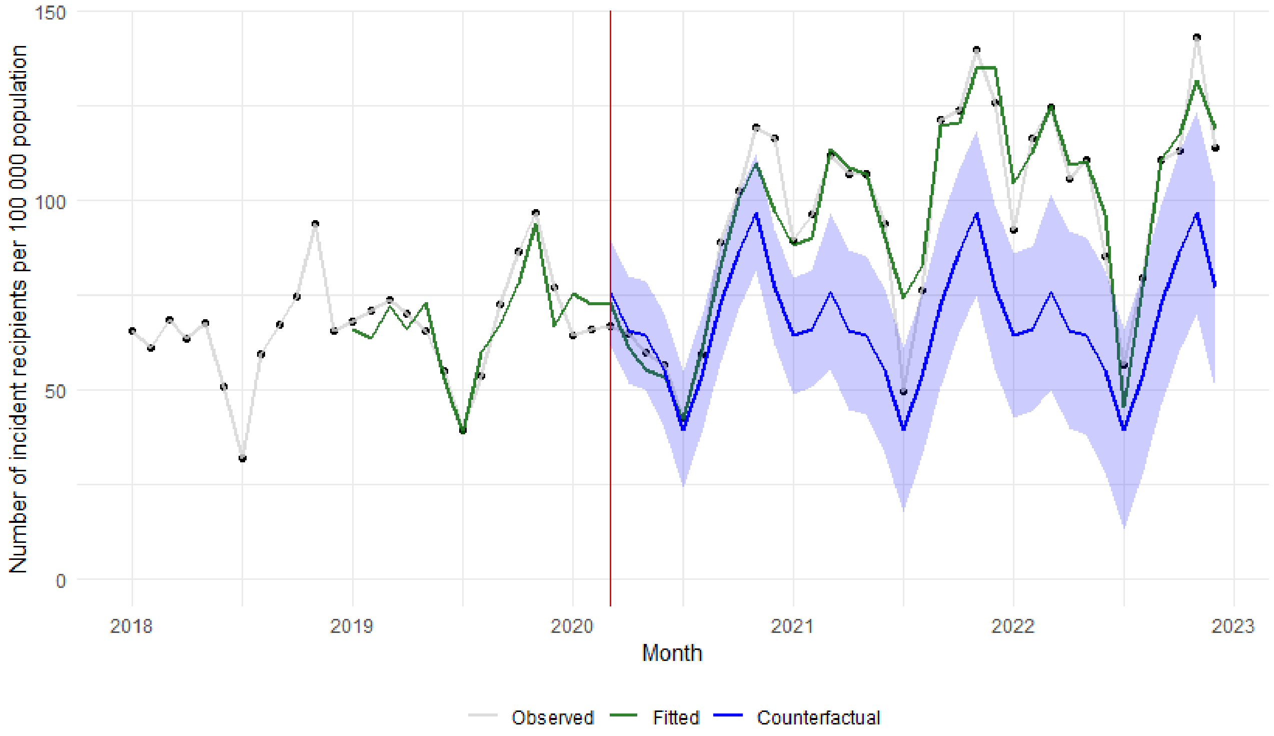

Fig. S35 ARIMA model of development in monthly incidence (per 100 000) in **males** in the age group 0–17 years, **Finland**

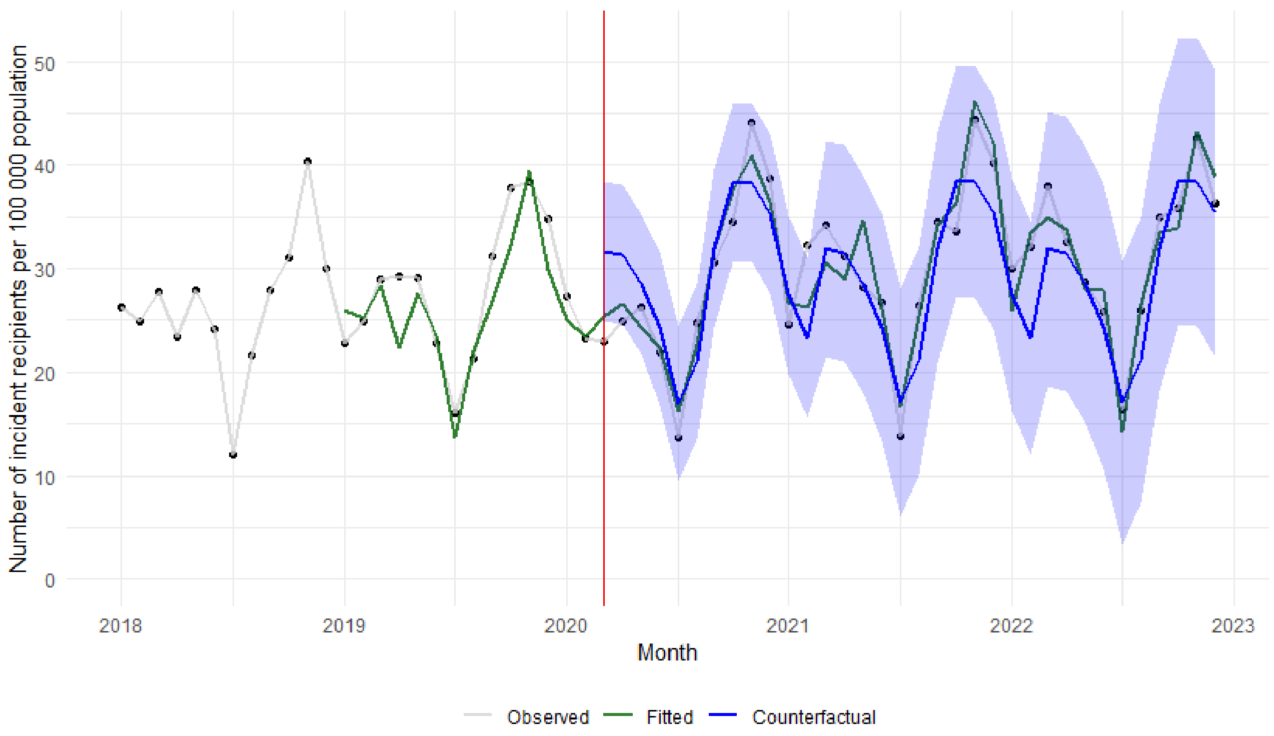

Fig. S36 ARIMA model of development in monthly incidence (per 100 000) in **females** in the age group 0–17 years, **Germany**

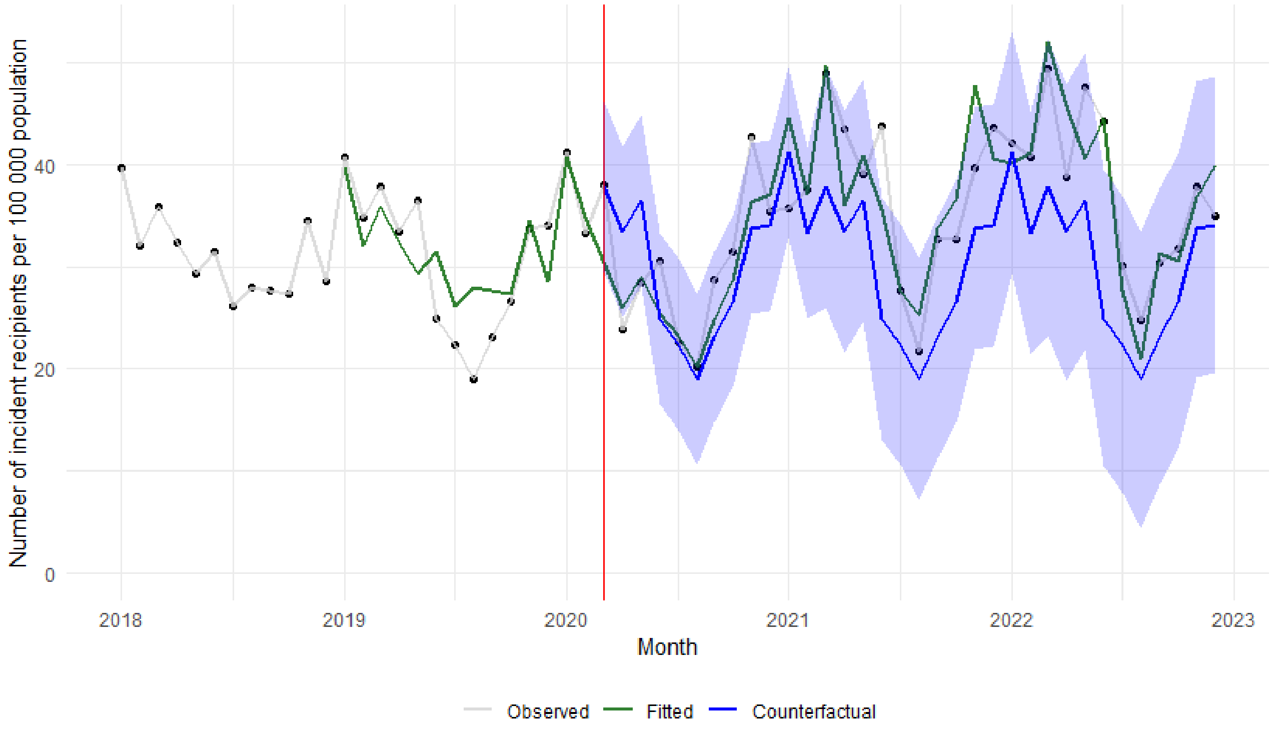

Fig. S37 ARIMA model of development in monthly incidence (per 100 000) in **males** in the age group 0–17 years, **Germany**

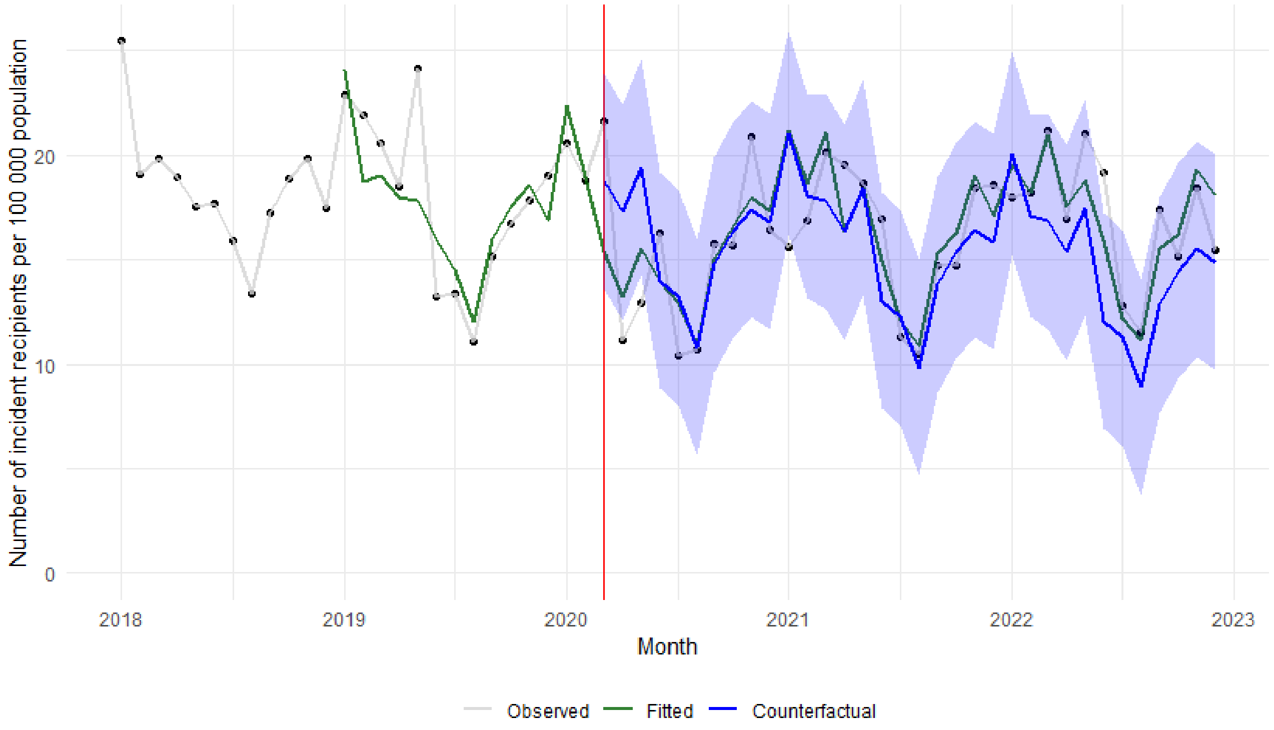

Fig. S38 ARIMA model of development in monthly incidence (per 100 000) in **females** in the age group 0–17 years, **Lombardy**

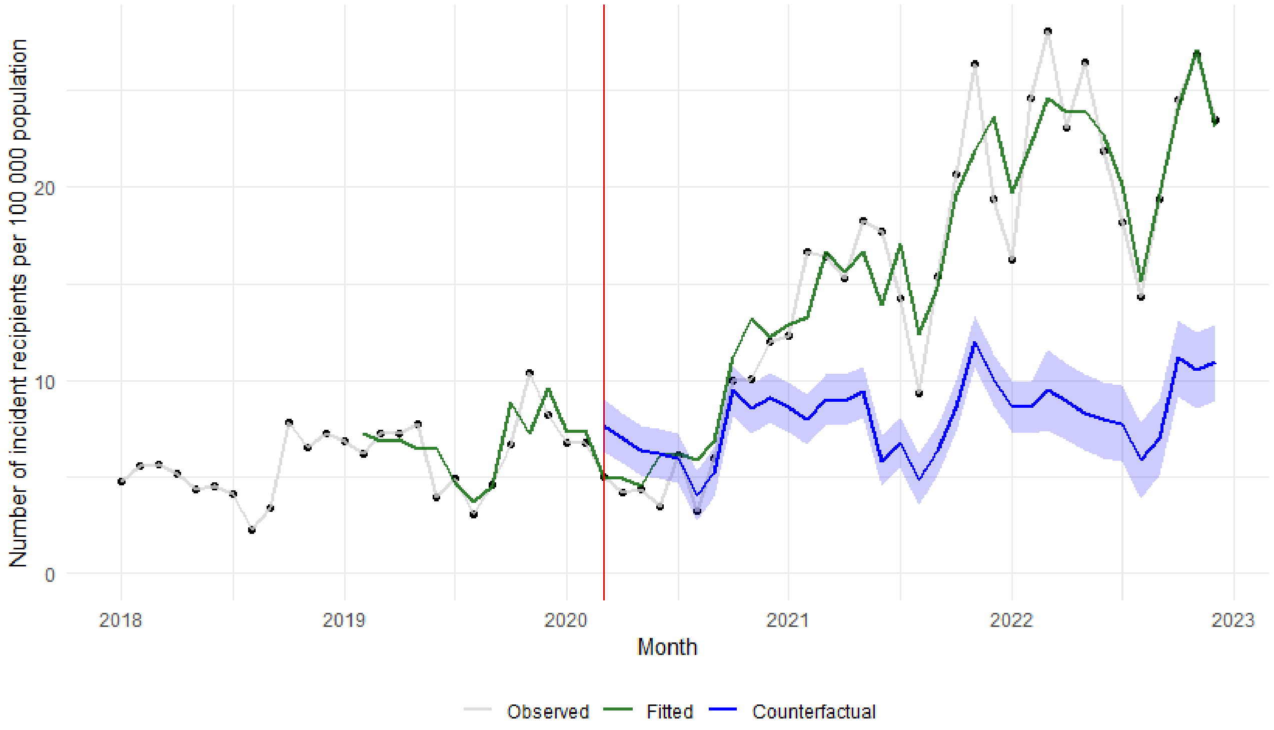

Fig. S39 ARIMA model of development in monthly incidence (per 100 000) in **males** in the age group 0–17 years, **Lombardy**

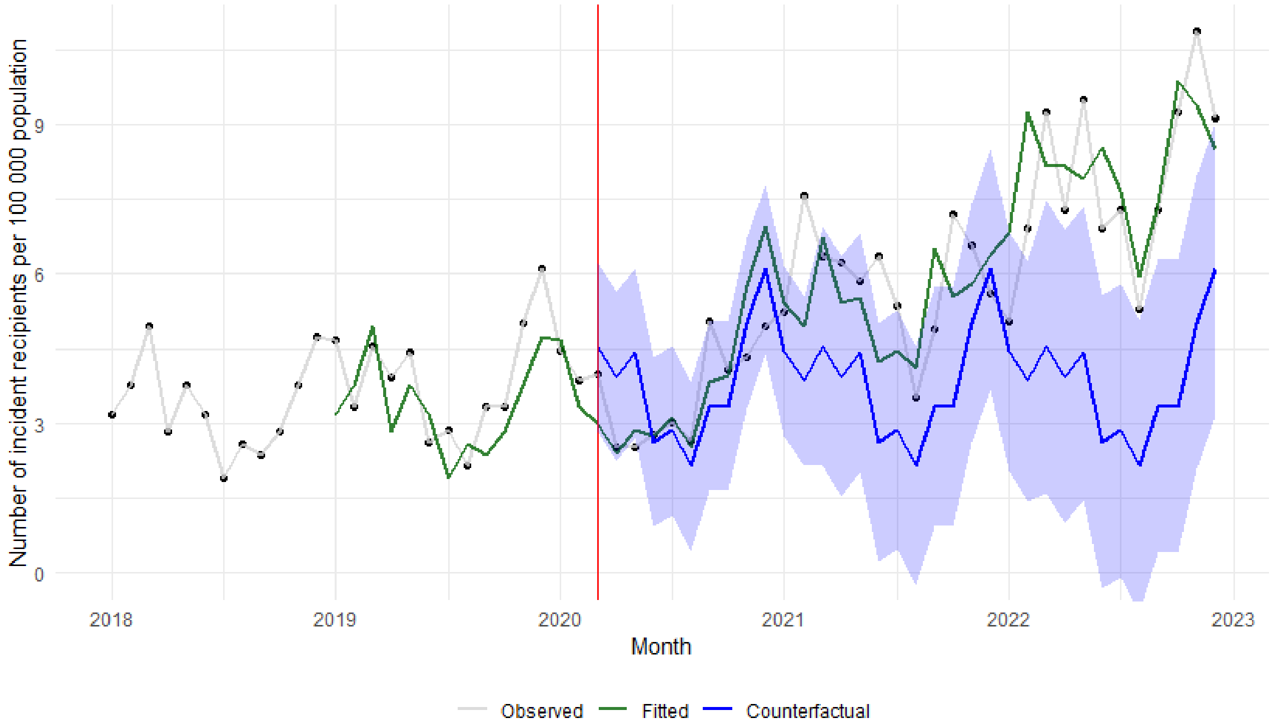

Fig. S40 ARIMA model of development in monthly incidence (per 100 000) in **females** in the age group 0–17 years, **Slovenia**

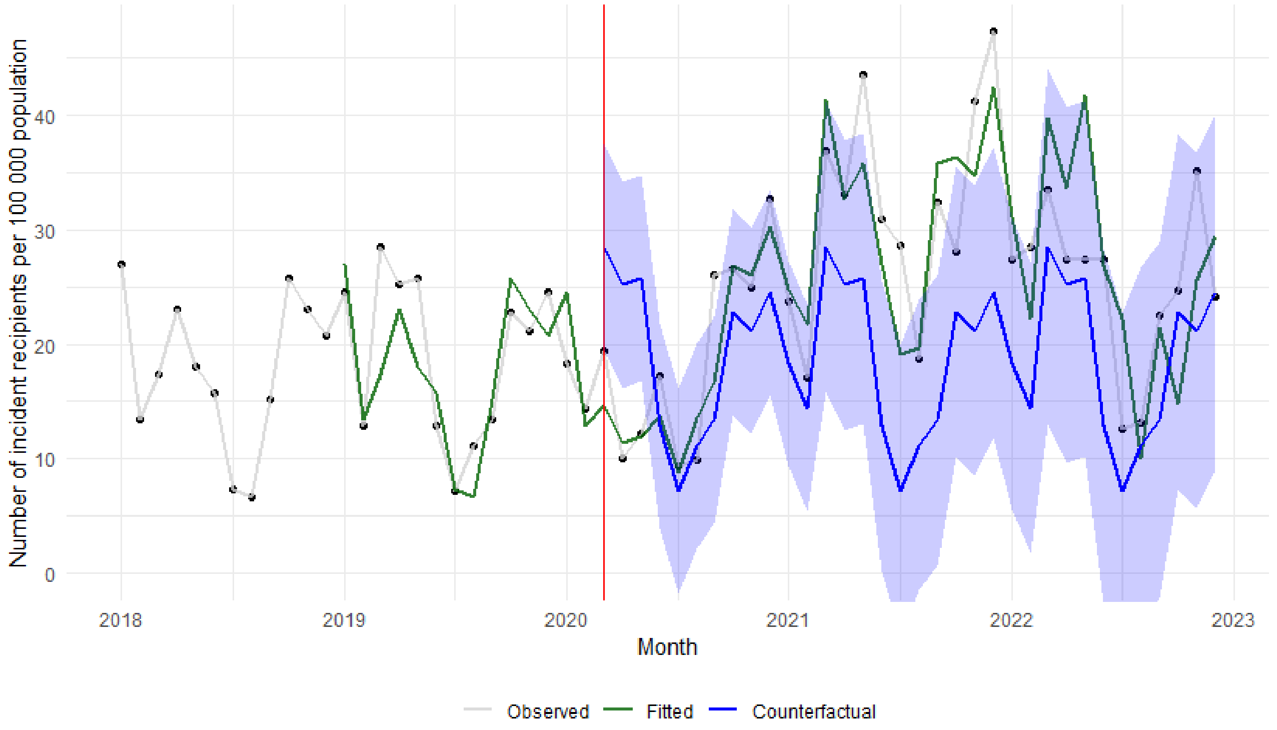

Fig. S41 ARIMA model of development in monthly incidence (per 100 000) in **males** in the age group 0–17 years, **Slovenia**

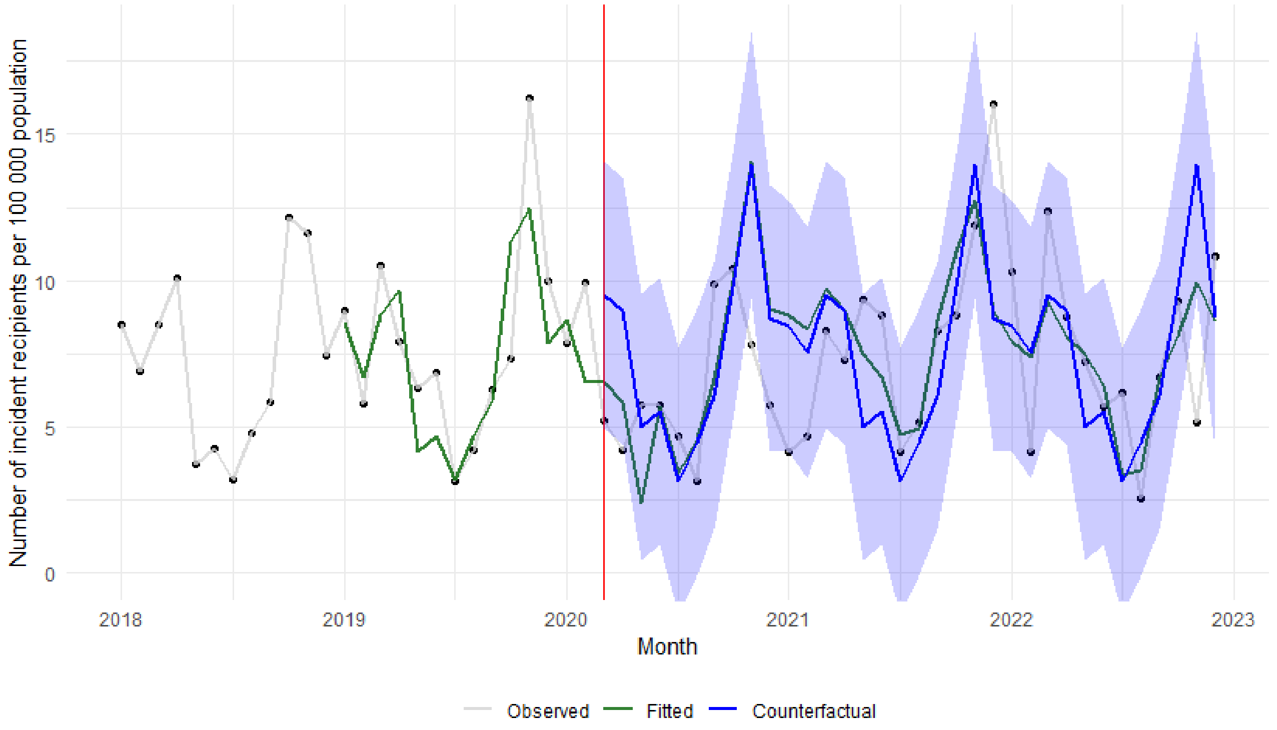

Fig. S42 ARIMA model of development in monthly incidence (per 100 000) in **females** in the age group 0–17 years, **Sweden**

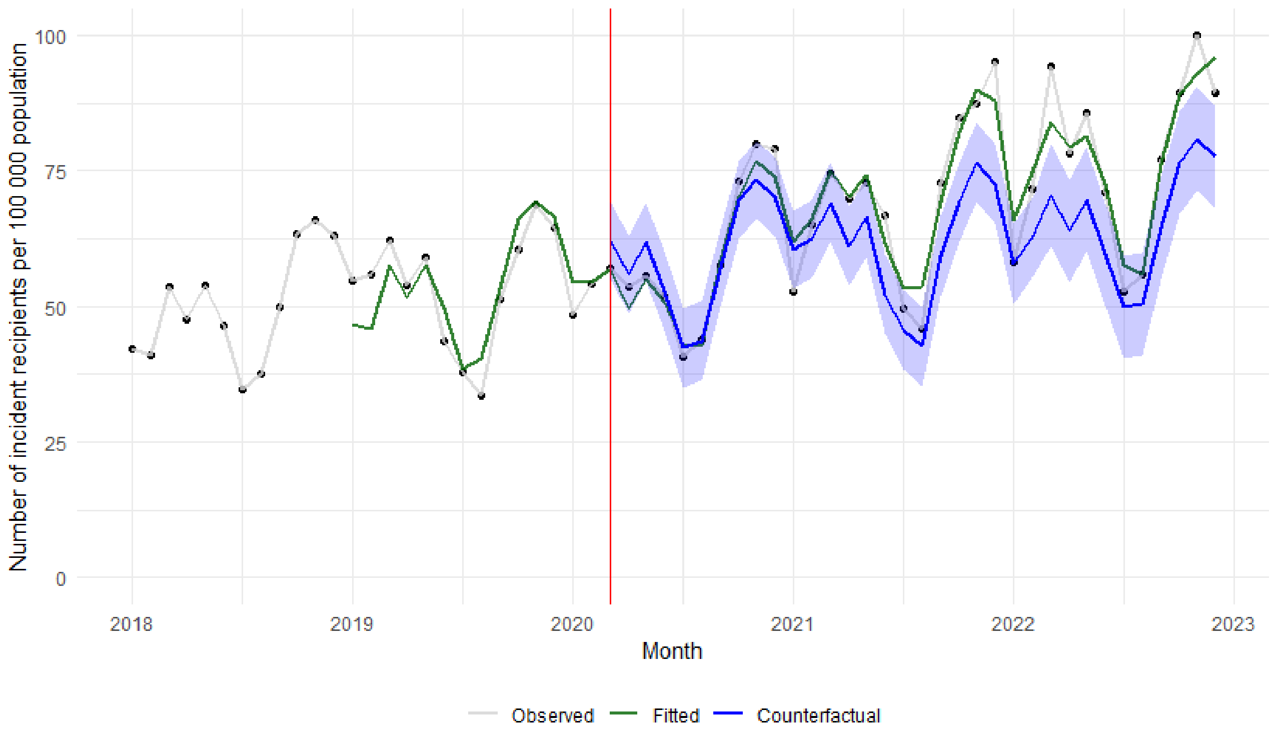

Fig. S43 ARIMA model of development in monthly incidence (per 100 000) in **males** in the age group 0–17 years, **Sweden**

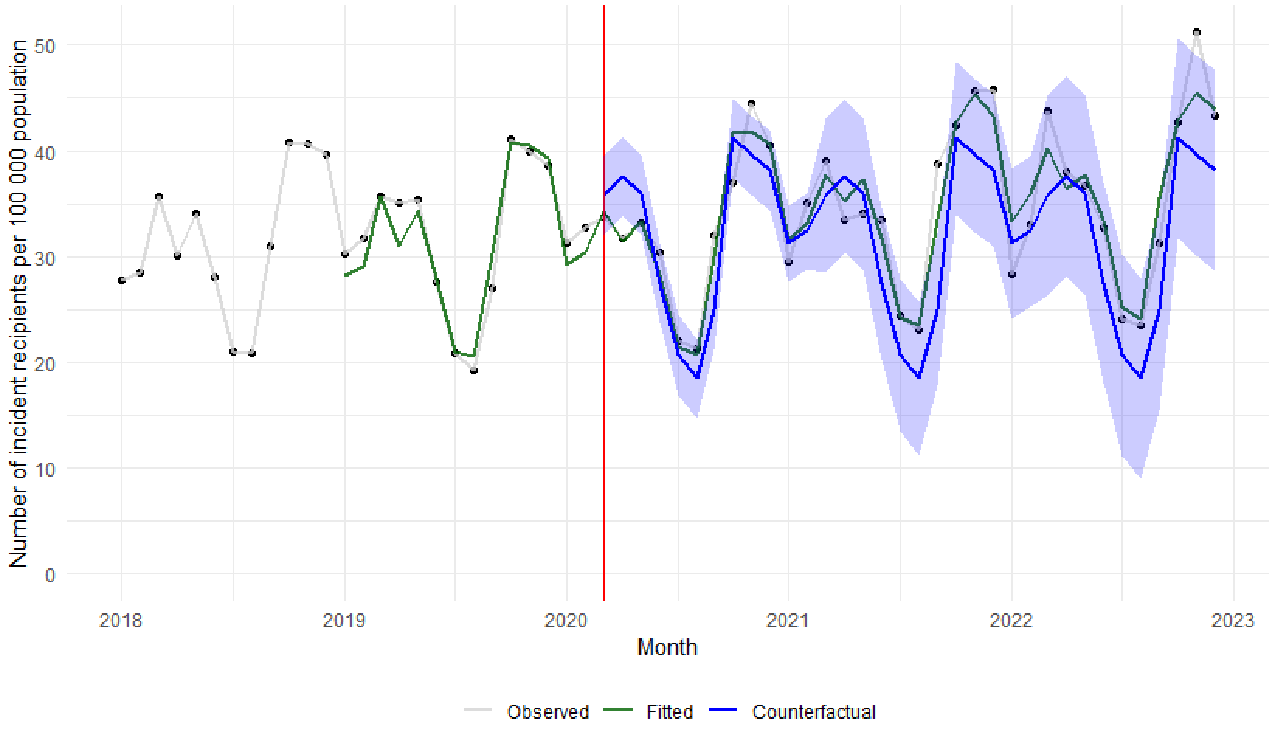

References

1. Reibling N, Ariaans M, Wendt C (2019) Worlds of Healthcare: A Healthcare System Typology of OECD Countries. Health Policy 123:611–620. https://doi.org/10.1016/j.healthpol.2019.05.001

2. NHS England (2024) NHS prescription charges. In: nhs.uk. https://www.nhs.uk/nhs-services/prescriptions/nhs-prescription-charges/. Accessed 16 Jul 2024

3. Ludwig W-D, Mühlbauer B, Seifert R (2024) Arzneiverordnungs-Report 2023. Springer

4. Institute for Health Metrics and Evaluation (2024) Global Burden of Disease Study 2019 (GBD 2019) Data Resources | GHDx. https://ghdx.healthdata.org/gbd-2019. Accessed 16 Jul 2024

5. Selke Krulichová I, Selke GW, Bennie M, Hajiebrahimi M, Nyberg F, Fürst J, Garuolienė K, Poluzzi E, Slabý J, Yahni CZ, Altini M, Fantini MP, Kočí V, McTaggart S, Pontes C, Reno C, Rosa S, Pedrola MT, Udovič M, Wettermark B (2022) Comparison of drug prescribing before and during the COVID-19 pandemic: A cross-national European study. Pharmacoepidemiol Drug Saf 31:1046–1055. https://doi.org/10.1002/pds.5509
